# Biodegradable Meets Functional: Dual-Nozzle Printing of Eco-Conscious Parklets with Wood-Filled PLA

**DOI:** 10.3390/ma18132951

**Published:** 2025-06-22

**Authors:** Tomasz Jaróg, Mateusz Góra, Michał Góra, Marcin Maroszek, Krzysztof Hodor, Katarzyna Hodor, Marek Hebda, Magdalena Szechyńska-Hebda

**Affiliations:** 1 Faculty of Architecture, Cracow University of Technology, Warszawska 24, 31-155 Kraków, Poland; tomekja28@wp.pl (T.J.); katarzyna.hodor@pk.edu.pl (K.H.); 2 Faculty of Material Engineering and Physics, Cracow University of Technology, Warszawska 24, 31-155 Kraków, Poland; mateusz.gora@atmat.pl (M.G.); michal.gora@doktorant.pk.edu.pl (M.G.); marcin.maroszek@doktorant.pk.edu.pl (M.M.); marek.hebda@pk.edu.pl (M.H.); 3 ATMAT Ltd., Władysława Siwka 17, 31-588 Kraków, Poland; 4 NETZSCH (Netzsch Instruments Ltd.), Halicka 9, 31-036 Kraków, Poland; krzysztof.hodor@netzsch.com; 5 W. Szafer Institute of Botany Polish Academy of Sciences, Lubicz 46, 31-512 Kraków, Poland

**Keywords:** biodegradable composites, PLA and wood filament, sustainable development, mobile urban furniture ‘parklet’

## Abstract

In the face of accelerating urbanization and the growing demand for environmentally responsible materials and designs, this study presents the development and implementation of a modular parklet demonstrator fabricated using dual-material 3D printing. The structure integrates polylactic acid (PLA) and wood-filled PLA (wood/PLA), combining the mechanical robustness of pure PLA in the core with the tactile and aesthetic appeal of wood-based biocomposite on the surface. The newly developed dual-nozzle 3D printing approach enabled precise spatial control over material distribution, optimizing both structural integrity and sustainability. A comprehensive evaluation was conducted for developed filaments and printed materials, including optical microscopy, coupled thermogravimetry analysis and Fourier Transform Infrared Spectroscopy (TG/FTIR), differential scanning calorimetry (DSC), and chemical and mechanical resistance testing. Results revealed distinct thermal behaviors and degradation pathways between filaments and printed parts composed of PLA and PLA/wood. The biocomposite exhibited slightly increased sensitivity to aggressive chemical environments and mechanical wear, dual-material prints maintained high thermal stability and interlayer adhesion. The 3D-printed demonstrator bench and stools were successfully deployed in public spaces as a functional urban intervention. This work demonstrates the feasibility and advantages of using biocomposite materials and dual-head 3D printing for the rapid, local, and sustainable fabrication of small-scale urban infrastructure.

## 1. Introduction

Traditional material manufacturing methods, e.g., injection molding, casting, or Computerized Numerical Control (CNC) milling, are associated with high energy and resource consumption [1]. Three-dimensional (3D) printing technology is an alternative to conventional manufacturing methods [2,3]; it enables the production of individualized forms with unique structures and geometries, including internal voids. This results in lightweight yet durable components with controlled mechanical properties, offering promising applications in architecture [2,4,5]. Despite its numerous advantages, the Fused Filament Fabrication (FFF) process also presents certain limitations, including (1) a restricted range of materials (primarily thermoplastics) compared to other manufacturing techniques; (2) surface finish and detail precision that often fall short of what is achievable through conventional technologies; and (3) limited efficiency for large-volume production, rendering 3D printing more suited to low-volume manufacturing [6].

Although the availability of thermoplastic materials is expanding, their diversity remains limited in the context of carbon-neutral production [6,7]. Increasing attention is being directed towards biodegradable bioplastics produced from renewable green resources, such as polylactic acid (PLA), polyhydroxyalkanoates (PHA), polybutylene succinate (PBS), and polycaprolactone (PCL) [8,9,10]. Among all raw materials used in 3D printing, PLA accounts for the largest share, comprising 37.1% [11]. PLA is a synthetic, high-molecular-weight polymer produced from corn starch. Non-toxicity, non-irritation, high tensile strength, good ductility, appealing gloss and transparency, excellent biocompatibility, and biodegradability make PLA particularly suitable for 3D printing [11]. A characteristic feature of PLA is its relatively low processing temperature, typically between 180 and 220 °C, which facilitates its application in FFF technology. Although PLA usually demonstrates lower resistance to mechanical loading compared to other commonly used thermoplastics, such as acrylonitrile–butadiene–styrene (ABS) [12,13,14], newer grades of PLA (e.g., 3D870) exhibit improved mechanical properties, rheology, chemical quality, and the ability to manipulate crystallinity compared to older types (e.g., 4043D) [15].

An increasingly common approach involves the use of biologically derived additives in the form of fibers [6], which can replace a significant proportion of the plastic during 3D printing [16,17,18]. Natural fibers, such as wood, cotton, jute, flax, hemp, and sisal, are characterized by low density, stiffness, damping properties, bending strength, and resistance to corrosion [6]. They are more cost-effective than synthetic fibers due to local availability and lower transport and production costs. Dispersed fibers, introduced into the polymer matrix as short, uniformly distributed fragments, are easily integrated with the FFF process [19]. They play a crucial role in dimensional stability and preserving the desired mechanical properties of the composite structure [18,20,21]. Fibers increase stiffness, mitigate shrinkage during cooling, limit fatigue crack propagation, reduce dynamic damage, and enhance the thermal conductivity of composites [22,23,24,25]. The incorporation of just 1% of highly dispersed microcrystalline cellulose into a polymer matrix can increase fracture energy by 200% [26]. Bamboo and agave cellulose fibers improve thermal stability and tensile strength, due to good interfacial adhesion between the fibers and PLA associated with the relatively high hydration level of cellulose, pectin, and hemicellulose [27].

Recently, multi-material and dual-material printing using PLA and fiber-modified PLA has been developed, enabling the fabrication of structures with varied mechanical and functional properties. Moreover, biomass-based fillers enhance the properties of PLA and open new opportunities for sustainable 3D printing [28]. The research utilizes dual nozzles for alternating layer deposition, such as PLA and lightweight PLA (LW-PLA), enabling control over porosity, density, and strength through adjustments to nozzle temperature and infill density [29]. The sequence of layering different filaments determines tensile strength, fatigue resistance, and thermal properties [28,30,31]. PLA composites reinforced with wood fibers and poly(butylene adipate-co-terephthalate) (PBAT) exhibit improved elastic modulus and crystallinity, with optimal fiber content providing the best mechanical and processing properties [31]. Often, composites display enhanced properties beyond the sum of individual materials; for instance, PLA/thermoplastic polyurethane (TPU) core–shell filaments show significantly higher crack resistance compared to pure PLA or TPU composites, and their properties can be tuned by varying the core volume fraction [32]. Alternative approaches involve printing PLA composites with metals [33], graphite [34], or conductive carbon black/PLA composites alongside pure PLA polymer [35]. The alternating deposition of conductive and dielectric layers influences electrical and mechanical properties, enabling applications in electronics and energy storage [34,35,36]. Literature reviews emphasize that biomass fillers improve PLA properties and open new avenues for sustainable 3D printing [28]. Furthermore, multi-material printing facilitates the development of 4D structures capable of shape change in response to temperature [29,32,35,36,37].

However, the current challenges in adopting these materials include, among others, moisture absorption and dimensional changes (limited environmental resistance) and the low mechanical (wind and sand abrasion), chemical (environmental pollutants, dust, PM particles, and acid rain), biological (microorganisms, algae, and fungi), and physical (UV photodegradation and temperature cyclic changes—freeze/thaw and heat/cool) stability of end-products, particularly under variable climatic (outdoor) conditions [38,39]. Natural fibers may also degrade at elevated temperatures during production, resulting in diminished mechanical properties [40]. Furthermore, challenges in 3D printing with dispersed fibers include the strict control of the homogenous dispersion of fibers in filaments; extrusion parameters (temperature or pressure) are key to prevent void formation within the material structure [41]. Dispersed fibers can also agglomerate, adversely affecting the material homogeneity and mechanical performance of prints [42]. Therefore, the objective of the present study was to develop 3D technology that enables precise spatial control over material distribution and print structural integrity; optimize filament material composition and its production process; analyze the feasibility of implementing an advanced additive manufacturing process for PLA reinforced with wood fibers, focusing on the fabrication of small-scale architectural elements; and thus improve the properties of new dual-material large-scale printing (stability, mechanical strength, chemical durability, surface wear, and environmental resistance).

## 2. Materials and Methods

### 2.1. Materials

Polylactic acid (PLA), poly(butylene adipate-co-terephthalate) (PBAT), and wood fibers were used for the preparation of filaments and 3D printing [19].

PLA type 3D870 (NatureWorks, Blair, NE, USA) was selected as the base polymer. This PLA grade has been optimized by the manufacturer for applications requiring enhanced mechanical strength and improved thermal resistance. The developed filaments were benchmarked against those of a commercially available PLA filament (PLA Fiberlogy, Fiberlab Ltd., Brzezie, Poland).

PBAT, specifically Ecoflex F Blend C1200 (BASF, Ludwigshafen, Germany), was employed as a flexibilizer while maintaining the biodegradable nature of the composite. PLA and PBAT are often co-utilized in 3D printing filament production due to the synergistic benefits of improved tensile strength, elasticity, and crack resistance, along with enhanced biodegradability [43,44]. PBAT positively influenced the processability of the blend, facilitating easier printing and increased resistance to environmental conditions [45,46].

Wood flour type CW630PU (JRS, J. Rettenmaier & Sohne GmbH, Rosenberg, Germany) served as an eco-friendly additive, reducing the proportion of synthetic polymers and enhancing mechanical characteristics such as stiffness and dimensional stability [47,48,49]. It also functioned as a filler, reducing shrinkage during printing and improving long-term structural stability. The average fiber length ranged from 20 µm to 40 µm. 

When selecting such materials for preparing filaments and 3D printing small-scale architectural models, several key criteria were considered, particularly a balance of mechanical strength, printability, environmental sustainability, and aesthetic appeal. A summary of the criteria for raw materials, along with the specific properties improved in composites, is presented in Table 1.

Although wood flour is typically added at up to 10% by weight to PLA, this study evaluated wood addition between 10% and 40%, with 20% yielding the most favorable properties (Table 2).

### 2.2. Filament Production

All raw materials were pre-dried for 24 h in a convection oven at 80 °C to eliminate residual moisture [19]. The filament production process comprised three main stages:

1. Shear and distributive mixing (homogenization) achieved using a laboratory-scale internal mixer (Haake PolyLab QC, Thermo Fisher Scientific Inc., Karlsruhe, Germany) at a rotor speed of 50 rpm and a temperature of 180 °C for 5 min. Torque feedback was integrated into the control loop to enable continuous process monitoring (a torque threshold of 35 Nm was maintained).
2. Granulate formation (pelletization) using a thermal die-face pelletizing unit (EUP 50, ECON GmbH, Weisskirchen/Traun, Austria). Specific granulation parameters are detailed in Table 3.
3. Continuous filament production using a co-rotating twin-screw extruder (TSK 30, Theysohn Extrusionstechnik GmbH, Stockerau, Austria) with a 20 mm screw diameter and a precision nozzle with a diameter of 2.8 mm. Specific extruder parameters are detailed in Table 3.

The extruded filament dedicated large-scale printing was conveyed onto a transport belt and spooled using a double-winding unit integrated into the SJC-45A extrusion line (Nanjing Tengda Machinery Co., Ltd., Nanjing, China), equipped with a 45 mm diameter twin-screw system and a L/D (length-to-diameter) ratio of 40:1. The extruder was equipped with five independently controlled heating zones, each regulated by electric resistance heaters and thermocouple feedback loops, enabling precise temperature control along the barrel. The temperature profile was set to increment progressively by 10 °C across the zones, ranging from 150 °C in the feeding zone to 200 °C at the die. The tension force of the filament winder was in the range of 10 N to 20 N. The extrudate was subsequently passed through a precision diameter control unit (±0.05 mm tolerance) and collected by a motorized puller and spooler operating at adjustable take-up speeds (5 m/min) to maintain dimensional stability. The extrusion process was monitored and controlled using PLC-based control software (version 5.1, Nanjing Tengda Machinery Co., Ltd., Nanjing, China) for the real-time adjustment of parameters.

Due to the high fiber content, the filament exhibited pronounced hygroscopic behavior that adversely affected the 3D printing process by bubble formation during the thermal decomposition of absorbed water. To mitigate this issue, the spooled filaments were subjected to post-extrusion drying at 80 °C for 24 h before printing (SpacePi X4 Filament Drying Box, Creality, Shenzhen, China) [19].

The validation of the process settings was considered when the produced filament met the following criteria: (1) The fiber remained properly embedded within the PLA matrix, with no fibers sticking out of the filament. (2) The filament diameter tolerance did not exceed ±0.03 mm. (3) The number of diameter reading errors was no more than one per 100 m of filament. (4) The number of air bubbles did not exceed one per 100 m of filament.

### 2.3. Optimization of Large-Scale 3D Printing Parameters

A series of experimental trials were performed using both PP and PPW filaments to set printing parameters and identify the optimal balance between (1) temperature and printing speed; (2) infill density; and (3) printhead utilization [19].

#### 2.3.1. Printing Temperature and Speed

The tested processing temperature ranged from 190 °C to 230 °C, exceeding the standard PLA range to accommodate the composite’s modified flow behavior. A bed temperature of 60 °C was maintained throughout all trials. The printing speed was maintained between 25 mm s^−1^ and 60 mm s^−1^, with the first layer printed at a fixed speed of 20 mm s^−1^ to optimize bed adhesion. To further enhance bed adhesion, the print surface was treated with an adhesion promoter (Dimafix, I3D Digital Media S.L., Almería, Spain). Cooling was initiated after the deposition of the second layer using a part-cooling fan operating at 50% of its maximum output (approx. 8–10 CFM airflow rate), corresponding to a PWM duty cycle of 50%. The fan was positioned laterally, directing the airflow at a 45° angle to the printed surface. This setting was calibrated to maintain a surface temperature of approximately 40–50 °C immediately after deposition, ensuring a balance between sufficient solidification and optimal interlayer bonding. Preliminary parameter calibration included temperature tower experiments and flow rate tuning to ensure dimensional accuracy and optimal interlayer adhesion [19].

#### 2.3.2. Dual-Printhead Deposition Strategy

**Figure 1:**
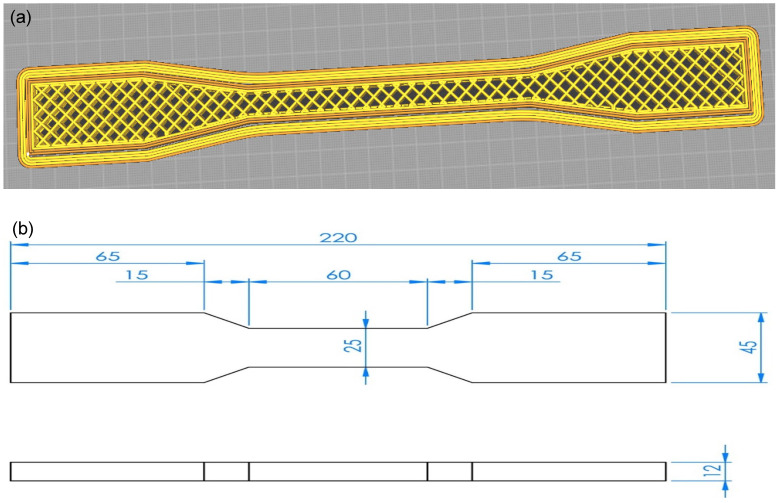
Specimen geometry (**a**) as defined in the slicing software UltiMaker Cura 5.10 (UltiMaker B.V., Geldermalsen, The Netherlands) and corresponding sample dimensions (**b**).

Test specimens were 3D printed following a modified ISO 527-2 Type 1A [50] geometry (Figure 1), tailored to reduce material usage. Three-dimensional printing was performed using an industrial ATMAT Saturn printer (ATMAT, Krakow, Poland), equipped with a four-zone heated build platform, enabling precise temperature control to improve adhesion. The printing chamber was thermally insulated and featured active temperature regulation to maintain a stable thermal environment during printing. Printing parameters included a layer height range of 0.2–1.0 mm. The nominal printing speed was set at 200 mm/s, with a high-quality mode operating at 100 mm/s. The maximum non-print travel speed reached 300 mm/s. Positioning accuracy was 50 µm for the X and Y axes and 10 µm for the Z axis, ensuring high dimensional precision. Print preparation and slicing were carried out using custom-developed software, Simplify3D version 5.1 (SIMPLIFY3D, Cincinnati, OH, USA), which facilitated the detailed control of printing parameters and support structures. Printing parameters for external outlines and infill regions are presented in Table 4.

A dual-printhead deposition strategy was employed by differentiating the printing parameters for external outlines (perimeters) and internal infill regions of samples. External perimeters were printed using the primary printhead, equipped with a smaller 1.0 mm nozzle. A standard layer height of 0.5 mm or 0.8 mm and a nominal material flow rate of 100% were applied (30 and 48 mm^2^ s^−1^, respectively) (Table 4 and Table 5). This configuration ensured high surface fidelity and dimensional accuracy, particularly in geometrically sensitive or visually exposed areas [19]. In contrast, the infill regions were printed with the secondary printhead and a larger 1.4 mm nozzle, assigned to layer heights ranging from 0.5 mm to 2.4 mm. A higher flow rate of 120–130% was applied periodically across the Z axis (ranging from 50.4 mm^2^ s^−1^ for 0.5 mm and 120% up to 262.1 mm^2^ s^−1^ for 2.4 mm and 130%) (Table 4 and Table 5). Specifically, for every one to three perimeter layers, a single infill layer with enhanced parameters was deposited. The variable perimeter-to-infill layer ratio (1:1 to 1:3) was selected based on the mechanical and structural demands of each component. The elevated flow rate in the infill region compensated for the higher volumetric extrusion required by the thicker layers, ensuring sufficient interlayer bonding, minimizing void formation, improving internal density, and preserving the surface quality and dimensional accuracy of the external perimeters [19].

#### 2.3.3. Urban Furniture Demonstrator ‘Parklet’

Building upon the outcomes of pilot-scale experiments, processing parameters were refined for the large-format additive manufacturing of an urban furniture demonstrator (“parklet”). The demonstrator was manufactured using the ATMAT Saturn large-format 3D printer (ATMAT, Krakow, Poland) and custom-developed software, Simplify3D 5.1, utilizing a double-printhead setup with a custom-designed wide hardened steel nozzle to prevent clogging by wood fibers and to optimize printing speed [19]. Representative printing parameters included a printing speed of 30 mm s^−1^, a flow rate of 100%, and nozzle temperatures ranging from 220 °C to 230 °C (Table 6). The optimal printing temperature for PPW (wood-filled composites) was 10 °C higher than for PP (unfilled PLA), attributed to the lower thermal conductivity and insulating properties of the embedded fibers [19].

**Figure 2:**
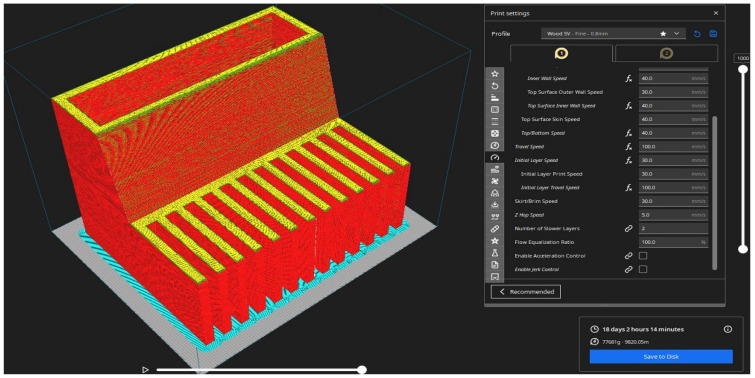
Three-dimensional printing slicer preview of a stepped overhang test model in PrusaSlicer (version 2.9.2, Prusa Research, Prague, Czech Republic). The model demonstrates various overhang angles to evaluate print quality under challenging geometries. The preview shows a color-coded representation of print speeds: red indicates faster print speeds, yellow indicates moderate speeds, and green indicates slower speeds. The model includes an array of horizontal overhangs above a solid base, with support structures (blue) generated below the overhang sections to ensure print stability. The right panel displays detailed print settings, including inner and outer wall speeds, infill speed, and support interface configurations.

In the final print using PPf and PPWf filaments, slightly modified parameters were applied for each printhead as part of a dual-extrusion strategy. This configuration was designed to ensure high print quality while allowing for the production of approximately 100 kg of printed components over an 18-day continuous printing period (Figure 2).

### 2.4. Sample Properties

Prior to thermal, chemical, and mechanical testing, all samples (filaments and 3D prints) were conditioned for 24 h at 23 ± 1 °C and 40 ± 5% relative humidity to facilitate the stabilization of residual thermal stresses and moisture content [19]. 

Untreated samples (a) were compared to samples treated 24 h at ambient temperature with the following chemical agents: (b) a sodium hypochlorite-based solution (approx. 2.5% NaOCl, Domestos, Unilever, London, Great Britain) to simulate oxidative stress; (c) a 1:1 volumetric mixture of concentrated nitric acid (65%, POCH Ltd., Gliwice, Poland) and hydrochloric acid (37%, POCH Ltd., Gliwice, Poland) used to test the resistance to strong inorganic acids; (d) mild abrasive paste (Polyboy, Lilienthal, Germany) containing 20% aluminum oxide (by weight) combined with organic oxalic acids at concentrations of 1–5% to test the impact of acidic organic matrices; (e) natural acidic media represented by freshly extracted clementine juice, rich in citric and ascorbic acids, used to simulate prolonged exposure to biologically derived weak acids; (f) iron gall ink (Parker, Nantes, France), to assess interactions with tannic acid and iron(II) sulfate; (g) an ethanol-based (70% EtOH, POCH Ltd., Gliwice, Poland) extract containing juglone (5-hydroxy-1,4-naphthoquinone), obtained from black walnut (*Juglans nigra*) husks (1:1 husks : EtOH), representing the class of natural quinones combined with alcoholic solvents; (h) hydrocarbon from a commercially available multipurpose lubricant (WD-40, Sandiego, CA, USA), composed primarily of petroleum distillates and aliphatic hydrocarbons; (i) pumpkin (*Cucurbita pepo*) seed oil (Oleofarm Ltd., Wroclaw, Poland), rich in unsaturated fatty acids, to assess long-term interaction with lipid-based organic compounds; (j) a 3% hydrogen peroxide solution (POCH Ltd., Gliwice, Poland) to simulate radical-induced polymer breakdown and oxidative degradation. For environmental stress testing, specimens immersed in (k) water were subjected to five freeze–thaw cycles at −20 °C, with each freezing phase followed by a full thaw at room temperature, in order to evaluate thermal expansion stress and microstructural resilience. Finally, mechanical abrasion resistance was tested by performing (l) five cycles of controlled surface friction using a standardized alumina-based grinding stone (grit size 80, Tormek AB, Lindesberg, Sweden) under constant load and duration.

The surface morphology and cross-sectional structure of both filaments and printed components were examined using a digital microscope (Keyence VHX-7000, KEYENCE International, Mechelen, Belgium).

Static tensile tests of the printed samples were conducted using the MTS Criterion Model 43 testing machine (MTS Systems, Eden Prairie, MN, USA), in accordance with ISO 527-1:2019 [51].

Thermogravimetric (TG/DTG) and Fourier transform infrared (FT-IR) analyses were carried out using a NETZSCH TG 209F1 Libra thermal analyzer integrated with an FT-IR spectrometer (NETZSCH-Gerätebau GmbH, Wittelsbacherstr. 42, D-95100 Selb, Germany). The measurements were conducted at a temperature ranging from 25 °C to 1000 °C and a constant heating rate of 10 °C min^−1^ under an argon atmosphere with a 20 mL min^−1^ flow rate. Infrared spectra were recorded in the wavenumber range of 600 to 4600 cm^−1^. Data were processed with the Opus 8.7 software (Bruker Optic GmbH, Ettlingen, Germany).

Differential scanning calorimetry (DSC) analysis was performed using a DSC 3500 Sirius instrument (NETZSCH-Gerätebau GmbH, Wittelsbacherstr. 42, D-95100 Selb, Germany), with samples placed in aluminum Concavus^®^ crucibles (NETZSCH-Gerätebau GmbH, Wittelsbacherstr. 42, D-95100 Selb, Germany). The measurements were conducted under a nitrogen atmosphere (99.99% purity), with a heating rate of 10 °C min^−1^, over a temperature range of 30 °C to 550 °C.

The reference (wood, cellulose, lignin, and hemicellulose) used for TG/DTG and DSC analyses has been published previously [52,53,54]. The reference spectra for FTIR peak assignments were obtained from the Opus 8.7 software (Bruker Optic GmbH, Ettlingen, Germany) database and relevant literature sources [55,56,57,58]. 

All measurements were performed with at least three repetitions for each set of materials (*n* = 3 to 5 for each print) and three technical repetitions for each type of analysis (n = 3 for chemical, mechanical sample treatment, and TG/DTG/FT-IR; *n* = 5 for tensile strength test and DCS; *n* = 10 for microscopic analysis). The repeatability of the result of measurement was below 5%.

## 3. Results and Discussion

### 3.1. Filament Properties

**Figure 3:**
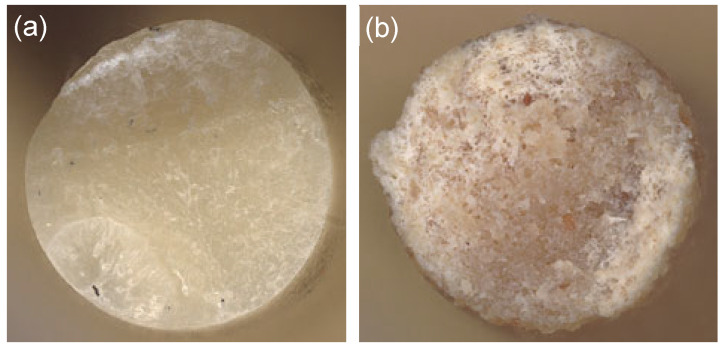
Cross-sectional view of the filaments PLA/PBAT: (**a**) wood-filled PLA/PBAT and (**b**) the structure and distribution of raw materials in the composite. Filament diameter: 2.8 mm.

Filament is a critical component in the Fused Filament Fabrication (FFF) printing process. Usually, wood flour is incorporated into polylactic acid (PLA) at concentrations of up to 10% by weight [47,48,49]. In this study, however, a novel processing method was developed for PLA composites modified with short, dispersed natural fibers at a much higher loading level. A prototype filament containing 40% wood fiber showed significant fiber agglomeration and caused frequent nozzle clogging, even when the nozzle diameter of the printer’s hot end was increased from 0.4 mm to 0.8 mm. Similarly, the composite containing 30% wood flour exhibited pronounced brittleness and poor processing stability; it fractured at 80 °C during post-extrusion drying and failed at multiple points on the wound spool. In contrast, the filament containing 20% wood flour (CW630PU, JRS) and 32% PBAT as a flexibilizer [43,44] (PLA/PBAT/wood = 3:2:1.25) demonstrated adequate FFF printing behavior and was successfully used to produce specimens for mechanical testing [19] (Figure 3). These composites are denoted as filaments PPWf1 to PPWf4 and printed samples PPWp1 to PPWp4. The addition of wood flour, a renewable and environmentally friendly material, not only reduced the proportion of synthetic polymers but also enhanced key mechanical properties, including stiffness and dimensional stability [47,48,49] when compared to materials consisting solely of PLA and PBAT (PLA:PBAT = 3:2), denoted as filaments PPf1 to PPf4 and samples PPp1 to PPp4. Furthermore, wood flour acted as a filler, decreased shrinkage during printing and improved structural stability during the end-use phase.

### 3.2. Printing Optimization

In the subsequent phase of the study, 3D printing parameters were optimized, and their influence on the quality and performance of printed specimens (at small and large scales) was evaluated. The developed PPf and PPWf were benchmarked against those of a commercially available PLA filament (PLA Fiberlogy). For commercial PLA, the recommended extrusion temperature was 190–220 °C.

For an industrial 3D printer (ATMA Saturn) and a dual-printhead deposition strategy applied to small-scale samples, the PPf and PPWf filaments did not fully melt at lower temperatures (190–200 °C). This resulted in extrusion interruptions, poor interlayer adhesion, and ultimately brittle printed parts. Optimal PPp (print) quality was achieved above 205 °C; small samples exhibited strong interlayer bonding and a defect-free surface finish. At higher temperatures, filament stringing and uncontrolled flow became evident, with these effects being markedly more severe in the PPWf composites due to their wood fiber content. Additionally, prints made with PPWf at elevated temperatures displayed an overly glossy surface, indicative of the thermal degradation of PLA in PPWp [19]. It was determined that 215 °C represented the upper threshold for producing prints of acceptable quality using PPWf and a 3D printer (ATMA Saturn).

For a large-format urban furniture demonstrator (“parklet”), the ATMAT Saturn large-format 3D printer was equipped with a custom-designed double-printhead setup, including a wide hardened steel nozzle to prevent clogging by wood fibers and to optimize printing speed. The optimal printing temperature for PPf and PPWf filaments was established in the range of 220 °C to 230 °C. This range provided the best balance between material flow, interlayer bonding, and minimal stringing, ensuring high-quality, dimensionally stable PPp and PPWp prints [19].

The printing speed for the first layer was reduced to 20 mm·s^−1^ to promote proper adhesion. Subsequent layers were printed at a speed of 25–60 mm·s^−1^. High-performance prints were received at a printing speed of 40 mm·s^−1^ and even up to 60 mm·s^−1^ for small samples, while this was 30 mm·s^−1^ for large-format urban furniture demonstrators.

### 3.3. Print Properties

**Figure 4:**
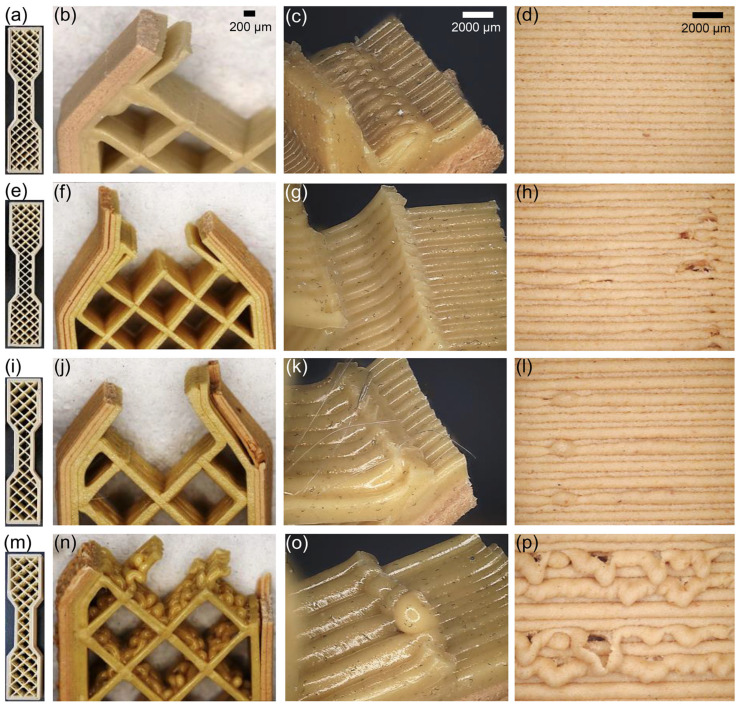
Samples printed with a dual-extruder strategy to receive different external outlines (PPWp1–PPWp4) and internal infill configurations (PPp1–PPp4). PPWp1/PPp1 (**a**–**d**), PPWp2/PPp2 (**e**–**h**), PPWp3/PPp3 (**i**–**l**), PPWp4/PPp4 (**m**–**p**) specimens prepared for tensile strength testing (**a**,**e**,**i**,**m**); fracture surfaces of the samples after tensile tests, indicating different failure modes depending on material composition and infill structure (**b**,**f**,**j**,**n**); close-up views of selected fracture zones, highlighting interlayer adhesion and fiber–matrix interaction (**c**,**g**,**k**,**o**); detailed images of printed layers in cross-section, with visible layer lines and occasional defects such as voids or poor bonding (**d**,**h**,**l**,**p**).

Four component variants were printed, each differing in geometrical parameters, with the infill density limited to a maximum of 25% (Figure 4). This constraint significantly improved printing efficiency and cost-effectiveness while preserving acceptable mechanical performance. Samples were printed using different external outlines (PPWp1–PPWp4, PLA/PBAT with 20% wood flour) and internal infill configurations (PPp1–PPp4, PLA/PBAT), implemented via a dual-extruder strategy. The objective was to assess the impact of outline-to-infill ratios and layer thickness on internal structure quality, interlayer bonding, and potential adhesion defects. In the balanced 1:1 configuration (PPWp1: 0.5 mm outline/PPp1: 0.5 mm infill), microscopic cross-sections confirmed effective bonding between the outline and infill regions and minimal voids, despite the use of two distinct materials. Fracture analysis of sample PPWp1/PPp1 revealed features typical of brittle failure (Figure 4a–d), consistent with the behavior of dry-state PLA under low-elongation conditions [59,60]. Increasing infill layer thickness to 1.0 mm (PPp2), while maintaining a thinner outline (PPWp2), led to the first signs of bonding imperfections at the interface. Sample PPWp2/PPp2 displayed mixed fracture characteristics, i.e., brittle regions alongside ductile zones with localized stretching and fibrillation (Figure 4e–h), indicative of heterogeneous filler effects such as wood particles in PLA [61,62]. Incomplete bonding was particularly evident near material transition zones. In the 1:3 ratio (PPWp3 outline: 0.5 mm/PPp3 infill: 1.5 mm), internal cohesion was reduced. Sample PPWp3/PPp3 exhibited a ductile fracture mode with elongated fibrils and micropores (Figure 4i–l), suggesting considerable plastic deformation capacity [61,62,63,64]. However, gaps, deformations, and poor interfacial bonding pointed to reduced thermal diffusion and mechanical integration at higher infill volumes and wider outline spacing. The most extreme configuration, with the thickest infill layer (PPp4: 2.4 mm) and thicker outline (PPWp4: 0.8 mm), showed severe defects—discontinuities, voids, and porosity-stemming from insufficient heat transfer and limited interlayer fusion. Sample PPWp4/PPp4 also demonstrated mixed fracture behavior, with predominant brittle zones and isolated regions of plastic deformation (Figure 4m–p), typical of composites where the rigid wood phase restricts plastic flow, while the PLA matrix retains some ductility [61,62,63,64].

Overall, increasing infill thickness and applying sparse outline-to-infill ratios degraded microstructural uniformity and cohesion. The most favorable structural properties were achieved in sample PPWp1/PPp1, where thin, evenly distributed layers promoted uniform bonding. Samples PPWp3/PPp3 and PPWp4/PPp4 delineate the processing limits for infill thickness and outline sparsity in maintaining structural integrity.

The lowest tensile strength and one of the highest elongation values were recorded for sample PLAp1, indicating limited mechanical resistance of the sample (Table 7). A slightly higher tensile strength at the same elongation was recorded for sample PLAp2, suggesting a partial improvement in mechanical properties. The obtained results correspond well with the mixed fracture characteristics described above. The highest tensile strength was observed for sample PLAp3, whereas elongation values were lower compared to the two previously described results. This is due to the distinctly ductile nature of the fracture, where the material could absorb significant stresses, but local elongation prior to fracture was limited [61,62,63,64]. Sample PLAp4, on the other hand, exhibited high mechanical strength with the greatest variation in results and the lowest elongation. The heterogeneous structure contributed to local stress and earlier crack initiation [65,66].

### 3.4. Chemical and Mechanical Properties of Filaments and Prints

**Figure 5:**
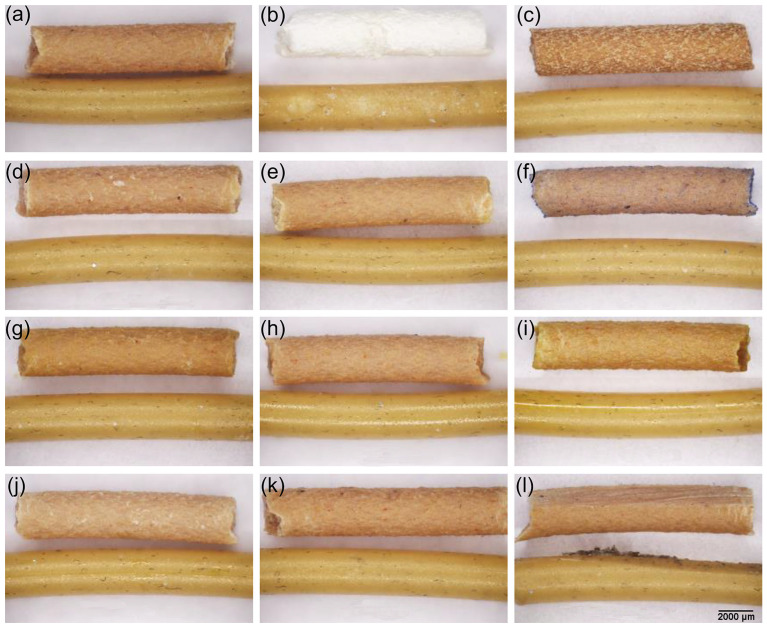
Morphology of PPWf (upper part of each photo) and PPf (bottom part of each photo) filament untreated (**a**) and treated with the chemical agents: (**b**) 4–5% NaOCl, (**c**) 65% nitric acid and 37% hydrochloric acid (1:1), (**d**) 20% aluminum and 2.5% organic oxalic acids, (**e**) natural citric and ascorbic acids, (**f**) tannic acid and iron(II) sulfate, (**g**) an ethanol-based extract containing juglone (5-hydroxy-1,4-naphthoquinone), (**h**) aliphatic hydrocarbons, (**i**) natural oil, and (**j**) 3% hydrogen peroxide, as well as (**k**) subjected to five freeze–thaw cycles at −20 °C and (**l**) mechanical abrasion (surface friction). Optical microscopy was applied to assess surface morphology. Marker: 2000 µm.

It has been shown that PLA composites demonstrate notable chemical resistance; however, the short-term chemical and physical effects, particularly under hygrothermal conditions, can induce potential brittleness [67]. In our study, the post-treatment evaluation of both filament and printed specimens revealed notable differences in chemical and mechanical resilience depending on material composition (Figure 5) [66,68,69]. Among the tested samples, the wood-based filament PPWf demonstrated higher sensitivity to chemical treatment under exposure to 4–5% NaOCl (Figure 5b). Pronounced whitening, surface softening, and visible deformation (e.g., tweezer indentation at the filament center) were observed. These effects were attributed to the heterogeneous, fibrous nature of the wood-based filler, which is more susceptible to hydrolysis and swelling [70,71]. In contrast, the same treatment induced only slight surface discoloration on the PPf surface. Treatments with concentrated acids (Figure 5c,d) but not natural acidic media (Figure 5e) led to the superficial roughness or dulling of PPWf samples. The application of natural dyes can also reveal differences in material affinity [69,72]. While iron gall ink, rich in tannins (Figure 5f), imparted a faint coloration to PPWf surfaces, the juglone-containing ethanol extract (Figure 5g), lubricant, oil, and hydrogen peroxide (Figure 5h–j) had no observable effect on either material. After environmental stress testing, specimens subjected to five freeze–thaw cycles (Figure 5k) did not break in terms of microstructural integrity. Finally, mechanical abrasion (Figure 5l) led to the partial removal of PPWf surface layers, resulting in localized fiber damage; in contrast, PPf showed only minor superficial damage but exhibited fiber pull-out and increased surface roughness.

**Figure 6:**
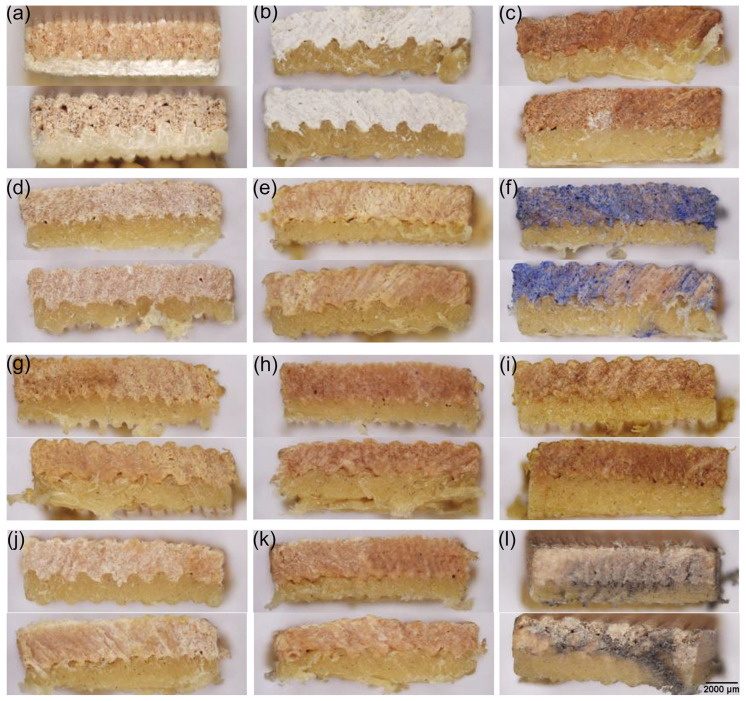
Morphology of PPWp (upper part of each sample) and PPp (bottom part of each sample) prints in combination PPWp1/PPp1 (upper part of each photo) and PPWp4/PPp4 (bottom part of each photo) untreated (**a**); treated with the chemical agents: (**b**) 4–5% NaOCl, (**c**) 65% nitric acid and 37% hydrochloric acid (1:1), (**d**) 20% aluminum and 2.5% organic oxalic acids, (**e**) natural citric and ascorbic acids, (**f**) tannic acid and iron(II) sulfate, (**g**) an ethanol-based extract containing juglone (5-hydroxy-1,4-naphthoquinone), (**h**) aliphatic hydrocarbons, (**i**) natural oil, (**j**) 3% hydrogen peroxide, as well as (**k**) subjected to five freeze–thaw cycles at −20 °C, and (**l**) mechanical abrasion (surface friction). Optical microscopy was applied to assess surface morphology.

These trends were also mirrored in the printed specimens (Figure 6), particularly in regions with wood fibers. Importantly, no delamination between print layers, neither along the longitudinal nor transverse axes, was observed in any sample, even after aggressive chemical and mechanical treatments. This finding highlights the structural durability and interlayer adhesion strength of the printed materials, even in the presence of heterogeneous fillers or under chemically aggressive conditions.

Altogether, the wood-based filament (PPWf) and prints (PPWp) demonstrated some sensitivity to chemical exposure. In contrast, PLA (PP) showed only minor changes under aggressive treatment conditions. PLA is a biodegradable polymer [72,73,74,75]; thus, its structural integrity can be compromised when exposed to chemical and mechanical factors, especially when heterogeneous fillers are involved [66,68,69]. These findings align with broader efforts to improve the functional resilience of PLA-based materials [69], particularly under chemical and mechanical stress [66,68,73]. The introduction of organic agents is effective in the formation of a more stable crystalline phase during PLA processing, thereby improving heat resistance and dimensional stability [68]. On the other hand, when wood is incorporated into PLA, this has been shown to permanently alter the wood’s nanostructure in ways that inhibit fungal activity and decay, even after prolonged water leaching [73,74]. Printing with two different materials using dual extrusion makes prints more effective because it allows for the exploitation of each material’s unique properties in specific parts of the print. Using a stronger PLA for the interior and a wood-based filament for the exterior offers a balanced combination of durability, aesthetics, and eco-functionality. However, understanding how wood interacts with PLA is critical for designing durable, multifunctional biocomposites.

### 3.5. Thermal Properties of Filaments and Prints

Thermogravimetry analysis (TG and derivative DTG) coupled with Fourier transform infrared spectroscopy (FTIR; Gram–Schmidt mode) was applied to recognize structural and chemical details of PP and PPW composites. Generally, PLA undergoes intense pyrolysis reactions at temperatures between 300 and 400 °C [76,77]. PLA can be synthesized either through the direct polycondensation of lactic acid or via the ring-opening polymerization of lactide. The lactide exists as three distinct diastereoisomers: poly(L-lactide) (PLLA), poly(DL-lactide) (PDLLA), and poly(D-lactide) (PDLA) [78]. Therefore, the final thermal properties of PLA are highly dependent on the distribution of these units along the polyester chain, the nature of the end groups, and the polymer’s thermal history [79]. Similarly, the thermal decomposition behavior of cellulose, lignin, and other polymeric wood biomaterials is dependent on their chemical composition and stability [52,53,54,80]. 

**Figure 7:**
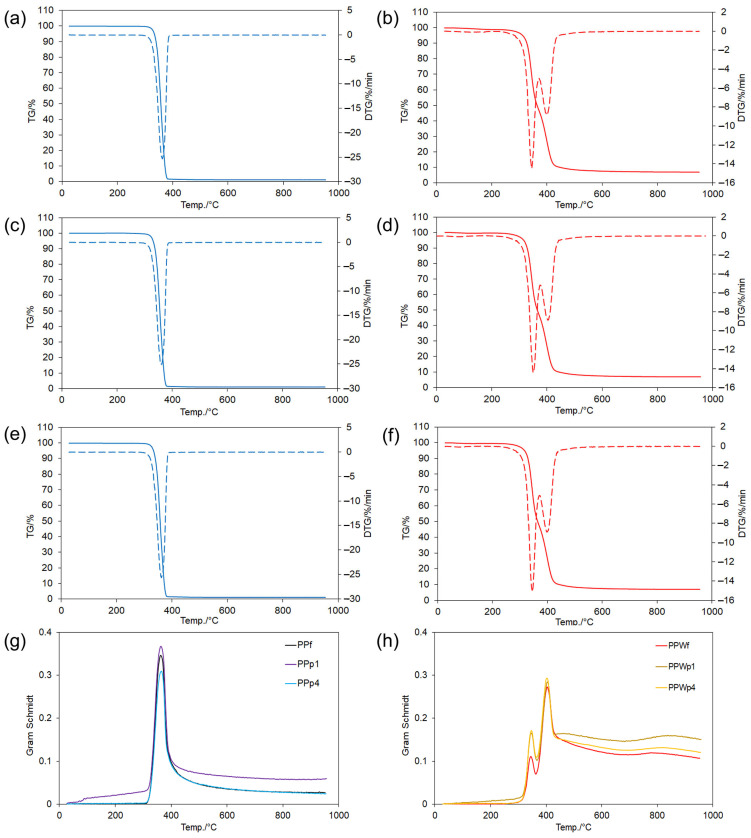
Thermal (TG and derivative DTG, **a**–**f**) and spectroscopic (FTIR Gram–Schmidt, **g**,**h**) analysis of filaments and printed samples. TG (solid line)/DTG (dotted line) curves were recorded for filaments (f) and prints (p): (**a**) PPf; (**b**) PPWf; (**c**) PPp1; (**d**) PPWp1; (**e**) PPp4; (**f**) PPWp4. Gram–Schmidt profiles were recorded for: (**g**) PPf, PPp1, and PPp4; (**h**) PPWf, PPWp1, and PPWp4.

Thermal effects and the corresponding evolution of gaseous products were observed for both PPf and PPWf (filaments) and PPp and PPWp (inner and external layers, respectively, in printed samples 1 or 4) across several temperature regions (Figure 7). Different behavior was related to PLA versus PLA/wood interactions [55,81].

At temperatures below 100 °C, the initial mass loss (TG/DTG, Figure 7), corresponding to the evaporation of water [52,54,80], was observed only for the PPWf and PPWp (<0.4%) but not for the PP samples. This was further supported by the absence of detectable decomposition products below 300 °C (Gram–Schmidt, Figure 7).

The thermal decomposition of all samples was initiated at 300 °C. Considering PP samples, a single pyrolysis stage occurred at 310–383 °C. The total mass loss exceeded 95% (TG—98.7% for PPf; 99.0% for PPp1; and 98.8% for PPp4). DTG showed only a slight variation under increasing temperature (25.5% min^−1^ at approximately 362.5 °C). However, Gram–Schmidt curves showed that the quantity of gaseous products increased in this order: PPp4 (0.31) < PPf (0.35) < PPp1 (0.37). DTG/TG and Gram–Schmidt curves often share extremum points during thermal degradation processes [82]. Therefore, less volatile gas evolved together with lower mass loss (98.8%) during PPp4 degradation, which might suggest that volatile degradation products are trapped or converted into solid char residues rather than being released (e.g., for pre-degraded PLA). PPW samples indicate improved thermal stability and two or three distinct pyrolysis stages within the range of 290–500 °C. The overall TG mass loss was lower (PPWf (93.1%) > PPWp1 (92.9%) > PPWp4 (92.8%)). In both the filament and printed samples, the first DTG peak exhibited greater mass loss (12.9% min^−1^ to 15.1% min^−1^ at 350 °C) compared to the second DTG peak (8.9% min^−1^ at 400 °C). The differences in the number of gaseous products released at 350 °C increased in the order of PPWf (0.111) < PPWp1 (0.165) < PPWp4 (0.170). An analogous trend was recorded at 400 °C, i.e., PPWf (0.272) < PPWp1 (0.284) < PPWp4 (0.293). An extra gaseous products were observed in the 450–550 °C range for all PPW samples. All thermal effects corresponded to wood components, with cellulose decomposition occurring around 350 °C and lignin decomposition taking place between 400 °C and 450–550 °C [53,54,80].

**Figure 8:**
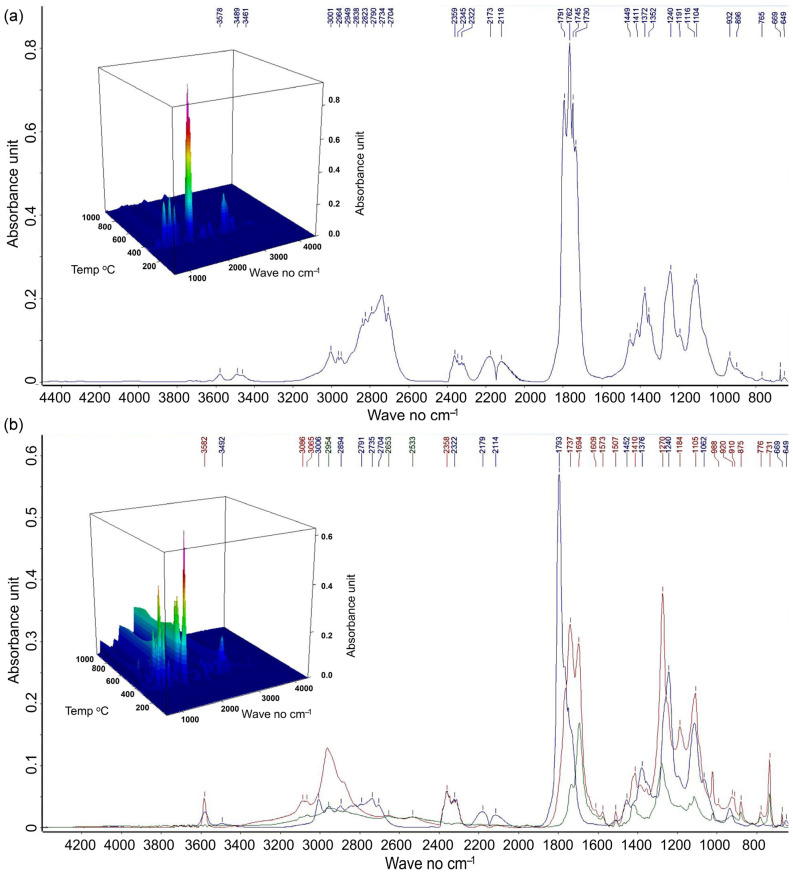
FTIR data in a 3D format with absorbance (arbitrary units, the color gradient indicates the magnitude of absorbance) as a function of both wavenumber (cm^−1^) and temperature (°C) for filament samples PPf (**a**) and PPWf (**b**), as well as 2D spectra extracted for the PPf (**a**) at 363 °C (blue line) and PPWf (**b**) at 348 °C (blue line), 407 °C (red line), and 787 °C (green line). The 2D spectrum for PPf (**a**) shows characteristic peaks related to the thermal degradation of PLA, i.e., 3578 cm^−1^: H_2_O, O–H stretching; 3489–3461 cm^−1^: water antisynchronous stretch, v3; 3100–2000 cm^−1^: organics, i.e., 3001 cm^−1^, 2964 cm^−1^, 2949 cm^−1^: CH_4_, C_2_H_6_, aliphatic C–H stretching; 2949 cm^−1^: C–H stretching, CH_4_; 2950–2650 cm^−1^ (mainly 2838 cm^−1^, 2823 cm^−1^, 2790 cm^−1^, 2734 cm^−1^, and 2704 cm^−1^): O–H, C–H stretching, corresponding to the stretching of methyl (–CH_3_) and methylene (–CH_2_–) groups; 2359 cm^−1^ and 2345 cm^−1^: CO_2_, C=O stretching; 2322 cm^−1^: C≡C stretching vibrations or CO_2_ absorption; 2173 cm^−1^ and 2118 cm^−1^: CO; 1762 cm^−1^: C=O stretching band, carboxylic acids; 1745 cm^−1^ and 1730 cm^−1^: C=O stretching, aldehydes, carboxylic acids, esters, and ketones; 1449 cm^−1^: C–H bending, CH_3_ and CH_2_ groups; 1411 cm^−1^: C–O stretch, carboxyl groups, O-H deformation, C–O–H deformation, COO− stretching, COO− ion; 1372 cm^−1^: C–H deformation, aliphatic compounds; 1352–1240 cm^−1^: C–H and =CH bending groups, C–O stretching, carboxylic acids; ~1200 cm^−1^ (1240 cm^−1^, 1191 cm^−1^): C–O–C stretching bands, aralkyl ethers; 1200–900 cm^−1^ (1116 cm^−1^, 1104 cm^−1^, 932 cm^−1^, 896 cm^−1^): C–O–C, C–O stretching; <900 cm^−1^ (896 cm^−1^): olefins, straight chain with seven or longer C atoms chains, epoxy rings, carboxylic acids, aromatic out-of-plane bend, C–O–C stretching; 765 cm^−1^: C–H bending and wagging vibrations; 669–649 cm^−1^: C–H bend (aromatic), alkenes, out of plane. The 2D spectrum for PPWf (**b**) shows characteristic peaks related to the thermal degradation of PLA (similar to those described for PPf, but with sifts towards decreased or increased wavenumber) and characteristic peaks related to the thermal degradation of wood, i.e., 3100–2995 (3086 cm^−1^): =C–H stretching, C–H aromatic chains stretch, C–H alkene chains stretch, lignin and C-H in PLA; 2894 cm^−1^ and 2653 cm^−1^: C–H asymmetric stretching (CH_3_, CH_2_); 2533 cm^−1^: carbonates; 1790 cm^−1^ and 1694 cm^−1^: C=O stretching (carbonyl), alkene, carboxylates; 1573 cm^−1^: C=C stretch aromatic compounds; 1507 cm^−1^: C=C stretching, aromatic skeletal, lignin, lignocellulose; 1270 cm^−1^: C–O–C stretching bands, C–O stretching (ester); 1184 cm^−1^: C–O–C asymmetric stretching (ester), phenolic; 1062 cm^−1^: C–O stretching, cellulose; 988 cm^−1^: C–O or C–C stretching, cellulose or PLA degradation; 920 cm^−1^: C–O–C ring stretching; 910 cm^−1^: skeletal vibrations; 875 cm^−1^: C–H bending (aromatic) for lignin; 776 cm^−1^: C–O–C stretching, amorphous cellulose, aromatic C–H bending of lignin and phenolic.

The recorded 3D FTIR spectra for filaments showed gaseous product evolution during thermal degradation up to 1000 °C (absorbance–wavenumber–temperature, as shown in Figure 8). Significant peaks appeared within the temperature range of 300–400 °C for the PPf sample, corresponding to PLA degradation [55]. An extended temperature range of 300–450 °C was recorded for the PPWf composite, which covers not only PLA but also extends to the main region of cellulose and lignin degradation [53,54,80]. The FTIR spectra were normalized relative to the same initial sample weight and underwent baseline correction. As a result, the overall integrated area under the curves reflected a higher amount of gaseous products evolved from PPWf [82,83].

In the 2D extracted FTIR spectra (Figure 8a), absorbance intensity highlighted seven principal regions (~3600, 3100–2600, 2400–2250, 2250–2000, 1850–1550, 1500–900, and 800–600 cm^−1^), corresponding to various PLA degradation products [55,56,57,58]. For PPWf samples, additional signals corresponding to cellulose and lignin degradation products were recorded, particularly at 348 °C, 407 °C, and 787 °C (Figure 8b).

The main gaseous products detected during raw PLA pyrolysis included CO, CO_2_, CH_4_, CH_3_CHO, esters, carbon-based compounds, ethane [11], organic compounds with carbonyl groups (mainly aldehydes, ketones, esters, and oligomers) [84], acetaldehyde, D,L-lactide, and meso-lactide [55]. In our research for PPf, the absorption region around ~3600 cm^−1^ corresponded to the stretching vibrations of –OH groups from water molecules (Figure 8). CO_2_ was detected in the region between 2400 and 2250 cm^−1^, while CO was identified at 2179 cm^−1^ and 2114 cm^−1^. More complex organic compounds were detected within the wavenumber ranges of 3100–2600 cm^−1^ (O–H and –CH_2_– stretching vibrations, with sharper peaks around 2800 cm^−1^ for aliphatic) and 1900–1700 cm^−1^ (C=O stretching vibrations of carboxylic acids, aldehydes, esters, and ketones). Between 1500 cm^−1^ and 800 cm^−1^, multiple absorption bands were recorded, related to C–H bending in CH_3_ and CH_2_ groups; C–O stretching in carboxyl groups; O–H and C–O–H deformation; COO^−^ symmetric and asymmetric stretching; =CH bending vibrations; and C–O–C stretching modes. Below 900 cm^−1^, signals corresponding to straight hydrocarbon chains with seven or more carbon atoms, epoxy rings, carboxylic acids, aromatic out-of-plane bending modes, C–O–C stretching, and C–H bending in aromatic rings and out-of-plane deformations typical for alkenes were observed.

PPWf composite exhibits a complex vibrational profile, indicative of the coexistence of polylactic acid (PLA) and lignocellulosic materials derived from wood. Several PLA-related peaks were observed to shift: 3578 cm^−1^ → 3582 cm^−1^, 3489 cm^−1^ → 3492 cm^−1^, 3001 cm^−1^ → 3006 cm^−1^, 2964 cm^−1^ → 2954 cm^−1^, 2790 cm^−1^ → 2791 cm^−1^, 2734 cm^−1^ → 2735 cm^−1^, 2359 cm^−1^ → 2358 cm^−1^, 2173 cm^−1^ → 2179 cm^−1^, 2118 cm^−1^ → 2114 cm^−1^, 1791 cm^−1^ → 1793 cm^−1^, 1730 cm^−1^ → 1737 cm^−1^, 1449 cm^−1^ → 1452 cm^−1^, 1411 cm^−1^ → 1410 cm^−1^, 1372 cm^−1^ → 1376 cm^−1^, and 1104 cm^−1^ → 1005 cm^−1^. Other peaks remained unchanged, e.g., at 2704 cm^−1^, 2322 cm^−1^, 1240 cm^−1^, 669 cm^−1^, and 649 cm^−1^. Several new peaks emerged, indicative of the thermal degradation of lignin at 3086 cm^−1^, 1573 cm^−1^, 1507 cm^−1^, 1184 cm^−1^, and 875 cm^−1^. In the spectral region between 1800 and 1300 cm^−1^, characteristic signals corresponding to organic acid anhydrides (1805–1770 cm^−1^), carbonate C–H vinyl groups and ketones C=O (1778–1770 cm^−1^), esters (1733–1725 cm^−1^), C=C vibrations (“fingerprinting” at 1502 cm^−1^), and C–H and =CH bending vibrations (1340–1339 cm^−1^) were also observed [53,54,80]. Other bands, including those at 2894 cm^−1^, 2653 cm^−1^, 2533 cm^−1^, 1270 cm^−1^, 1062 cm^−1^, 988 cm^−1^, and 776 cm^−1^, are indicative of thermal cellulose depolymerization and dehydration. Characteristic marker peaks were identified at 1200–1000 cm^−1^ and 1240 cm^−1^, corresponding to C–O stretching vibrations within the glucosic backbone; at 1105 cm^−1^, assigned to C–O–H alcohol groups; at 1452 cm^−1^, associated with C–H stretching in ethers; and at 689 cm^−1^, related to aromatic hydrocarbons. These signals support evidence of cellulose breakdown and the cracking of organic components into CO_2_ and CO [53,54,85]. In the cellulose “fingerprinting” region (1800–1500 cm^−1^, e.g., 1745 cm^−1^, [86]), a progressive decrease in the relative absorbance of peaks was also observed with increasing temperature (787 °C, 407 °C, and 348 °C; see Figure 8) confirming that cellulose degradation occurs primarily at 348 °C.

**Figure 9:**
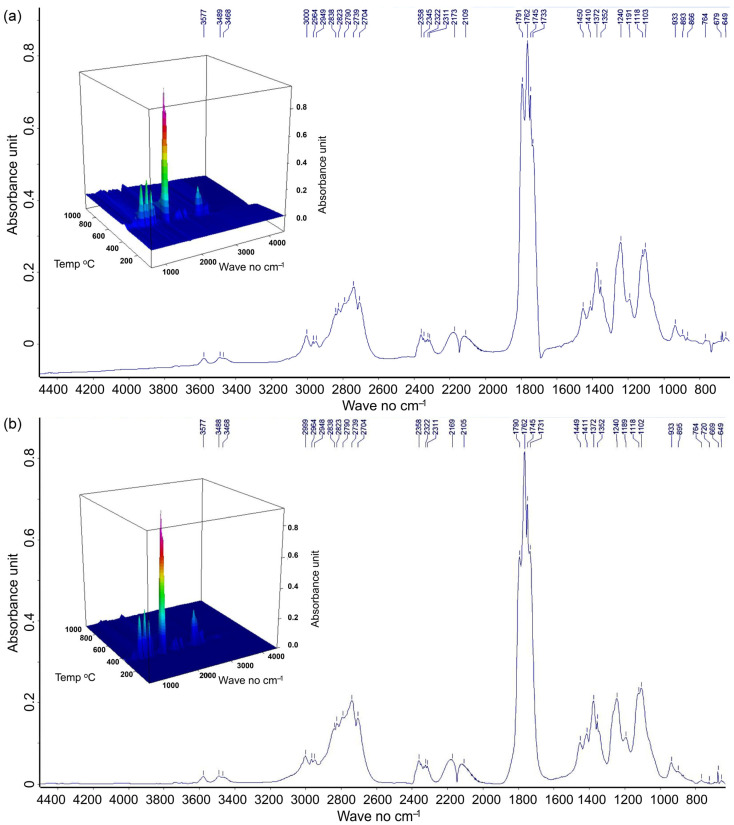
FTIR data in a 3D format with absorbance (arbitrary units, the color gradient indicates the magnitude of absorbance) as a function of both wavenumber (cm^−1^) and temperature (°C) for printed samples PPp1 (**a**) and PPp4 (**b**), as well as 2D spectra extracted for the PPp1 (**a**) at 363 °C and PPp4 (**b**) at 372 °C (blue lines). The 2D spectra for PPp1 (**a**) and PPp4 (**b**) exhibit characteristic peaks associated with the thermal degradation of PLA, showing high similarity to each other and to those described for PPf (Figure 8a).

The changes in relative absorbance of the marker peaks at 1737 cm^−1^ and 1694 cm^−1^ between the filament and the printed samples (Figure 8 and Figure 9) indicate structural and chemical alterations during the printing process of different samples [86,87]. The multifaceted interactions between the PLA matrix and the cellulose, hemicellulose, and lignin within the composite filaments [55,56,57,58] likely influence not only the mechanical properties and thermal behavior of the printed material but also its microstructural integrity and adhesion between phases. Indeed, in PLA/lignocellulose composites, the characteristic PLA peak at 1750 cm^−1^ (C=O stretching band) shifted to ~1745 cm^−1^ [88,89,90]. This shift, along with the broadening of the O–H stretching band and intensified cellulose peaks at 3333 cm^−1^ and 1040 cm^−1^, suggests interactions between PLA and the hydroxyl groups present in wood-derived cellulose [91]. Moreover, new peaks at 1507, 1184, and 988 cm^−1^, associated with lignin aromatic vibrations and cellulose incorporation [91], or peaks at 1573 cm^−1^ and 1270 cm^−1^, attributed to lignin aromatic C=C stretching and C–O stretching modes [92], have been reported as proof of successful fiber incorporation. On the other hand, the absence of PLA-related peaks such as 1762 cm^−1^, 1730 cm^−1^, and 1116 cm^−1^ [58,91] followed fiber incorporation, i.e., chemical interaction and partial structural rearrangement [93]. 

FTIR spectra of the printed PLA internal regions of PPp1 (correct layer prints) and PPp4 samples (characterized by the presence of bubble defects) revealed similar spectral profiles and thus comparable chemical composition (Figure 9). However, a slight shift in the carbonyl stretching band was observed from 1791 cm^−1^ in PPp1 to 1790 cm^−1^ in PPp4. This subtle shift may suggest minor differences in the crystallinity, influenced by the presence of internal stresses or structural defects [94,95]. Additionally, a significant decrease in absorbance intensity was detected at 1790 cm^−1^ (carbonyl C=O stretching) and 1240 cm^−1^ (C–O, C–O–C stretching vibrations) in PPp4 compared to PPp1. It implies a reduced dipole interaction or possible disruption in polymer chain ordering (reduced crystallinity) in the defective region within PPp4 [94,95].

**Figure 10:**
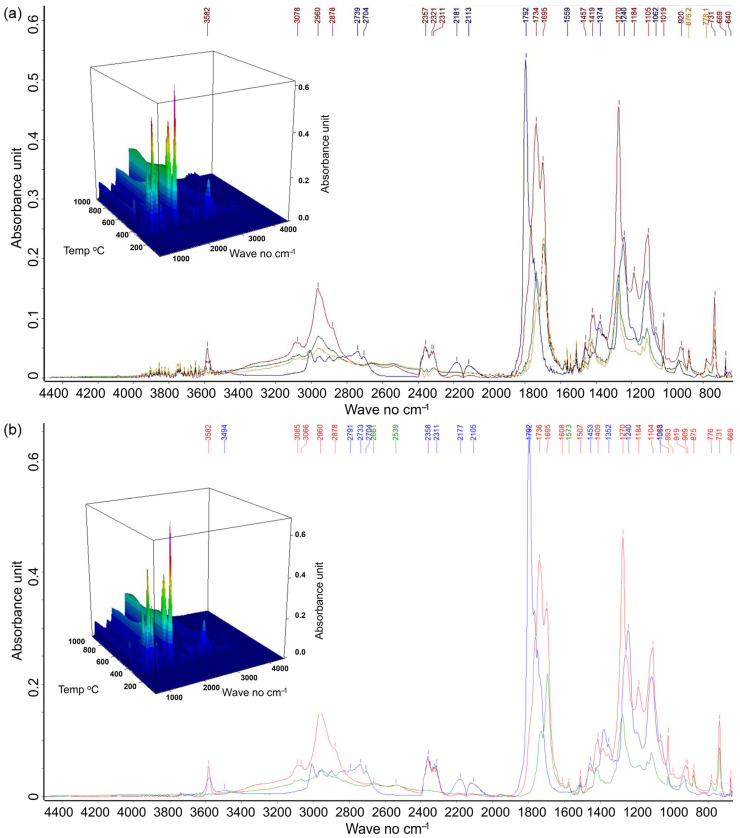
FTIR data in a 3D format with absorbance (arbitrary units, the color gradient indicates the magnitude of absorbance) as a function of both wavenumber (cm^−1^) and temperature (°C) for printed samples PPWp1 (**a**) and PPWp4 (**b**), as well as 2D spectra extracted for the PPWp1 (**a**) at 347 °C (blue line), 409 °C (red line), 470 °C (green line), and 859 °C (orange line), and PPWp4 (**b**) at 351 °C (blue line), 406 °C (red line), and 830 °C (green line). The 2D spectra for PPWp1 (**a**) and PPWp4 (**b**) exhibit characteristic peaks associated with the thermal degradation of PLA, showing high similarity to each other and to those described for PPWf Figure 8b).

The FTIR spectra of printed samples with wood fibers reveal few distinct spectral changes (Figure 10). In the 4000–3600 cm^−1^ region, the presence of numerous minor peaks suggests residual O–H stretching vibrations, which can originate from hydroxyl groups in cellulose and lignin of PPWp1 [55,81,96]. These weak signals, alongside the absence of the broad 3494 cm^−1^ peak (present in PPWp4), imply dehydration and hydrogen bonding or condensation reactions, leading to a more compact and less hydrophilic matrix. This supports stronger layer adhesion and reduced void formation during 3D printing. The pronounced peak at 2960 cm^−1^ (C–H stretching of methyl/methylene groups) with higher absorbance at 787 °C corresponds to secondary aliphatic changes due to lignin and hemicellulose thermal degradation. A reduction in absorbance at 1792 cm^−1^ (C=O stretching of carboxylic acids and esters) suggests a lower extent of random thermal degradation and fewer volatile acidic by-products [97,98]. This favored mechanical cohesion and avoided bubble formation during extrusion. Notably, an increased number of peaks in the 1500–1600 cm^−1^ region reflects the C=C stretching of aromatic rings (e.g., 1573 cm^−1^ and 1507 cm^−1^), typical of lignin degradation products [54]. It may have enhanced viscosity and interlayer diffusion, contributing to the smooth, continuous deposition in the PPWp1 sample. A stronger 1374 cm^−1^ peak at 363 °C, linked to C–H deformation in aliphatic chains, and the intensified 1270 cm^−1^ band (C–O–C and ester stretching) are consistent with the breakdown of PLA chains and stable ester functionalities, suggesting a more homogeneous degradation process. Meanwhile, lower absorbance in the 1300–1000 cm^−1^ region, especially at 348 °C, indicates the partial breakdown of polysaccharide (cellulose/hemicellulose) structures [54].

DSC (differential scanning calorimetry) provided a thermal analysis of filaments and printed samples. Minor fluctuations in the 50–200 °C region may be related to moisture evaporation or the rearrangement of amorphous regions in the polymers, particularly visible in the PPWp. Differences could also indicate changes in crystallinity or residual stress introduced during printing [99,100,101].

**Figure 11:**
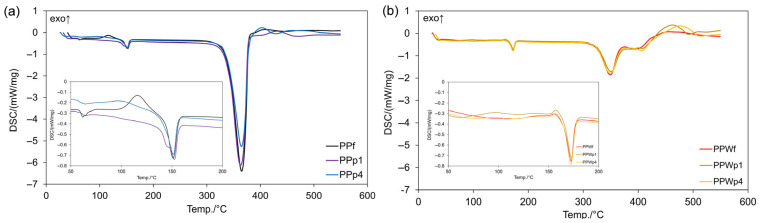
Differential scanning calorimetry thermograms (DSC, temperature range 20–600 °C) of (**a**) filament PPf and printed samples PPp1 and PPp4, as well as (**b**) filament PPf and printed samples PPp1 and PPp4. The inset highlights transitions in the lower temperature range (50–200 °C). The arrow and the label “exo” refers to exothermic processes.

A subtle baseline shift between ~55–65 °C (glass transition [101]) was visible in PPf and the internal part of prints (Figure 11), but the effect was lowered in this order: PPf > PPp4 > PPp1. A slightly broader and less defined transition of PPp1 and PPp4 indicated differences in chain mobility and thermal history of printing. The PPf filament also showed a more intense exothermic cold crystallization peak, related to a higher degree of amorphous content that crystallizes upon heating [102,103]. In contrast, both printed samples, especially PPp1, displayed reduced cold crystallization intensity, suggesting partial crystallization occurred during the printing process (to a lesser degree for PPp). All samples exhibited a melting peak in the ~160–170 °C range, though it appears slightly broader and less intense for the printed specimens. This could be attributed to structural differences induced by layer-by-layer deposition and thermal cycling during the 3D printing process.

The DSC analysis of PPWf and the external part of prints (Figure 11) revealed more complex thermal transitions. In the glass transition region (~55–65 °C), only a minor baseline shift was observed, indicating reduced chain mobility due to filler interactions or partial water retention in the hydrophilic wood phase, which plasticizes the matrix [101]. The cold crystallization peak (~110–140 °C) was notably weaker or absent, suggesting pre-crystallization occurred during extrusion or printing, facilitated by filler-induced nucleation and moisture-assisted molecular rearrangement [102,103]. The presence of bound or residual water in lignocellulose may also accelerate segmental motion and crystallization during cooling, reducing the amorphous fraction upon reheating. An additional broad endothermic signal below ~120 °C may be partly due to moisture evaporation, particularly in PPWf and PPWp4. This effect is likely due to the more hydrophilic nature of materials. The melting peak appeared broader and shifted to higher temperatures (165–170 °C), attributed to the heterogeneous crystallite structure of wood fibers and their uneven distribution.

All curves exhibit a sharp endothermic peak between 360 and 380 °C, characteristic of the thermal degradation of PLA. PPWf demonstrates a broader and multistep degradation profile, with an additional shoulder around ~400 °C. It was likely related to the decomposition of wood components (e.g., hemicellulose, cellulose, and lignin), increased filler–matrix interactions, and possibly residual degradation products from heating [52,53,54].

Overall, these observations confirm that the thermal history and degree of crystallinity in printed samples differ notably from those of the original filament. Additionally, variations in material composition significantly influence the final thermal properties. The printed parts, particularly those with higher perimeter counts or composite fillers, exhibit a pronounced shift toward pre-crystallized behavior and diminished thermal transitions. These changes have direct implications for the mechanical strength, dimensional stability, and thermal performance of the final printed components.

### 3.6. Development of the Parklet Demonstrator Using Dual-Material 3D Printing

The sustainable development and ecology are increasingly evident across various industrial sectors. Consequently, there is a growing need to develop new methods and materials that are less burdensome to the environment. As urbanization intensifies, focus increasingly shifts not only to the function and form of urban design but also to the materials used [104,105]. Small-scale, temporary structures like parklets, installed in parking spaces, promote public rest and interaction [106]. Traditionally made from wood, steel, or concrete, these structures are now often built with alternatives such as composites, recycled plastics, biopolymers, and plant-based materials [107,108]. Recycled plastics are valued for sustainability, especially when combined with plant fibers, which enhance durability and eco-performance [109]. Biocomposites offer a low carbon footprint and are lightweight and aesthetically pleasing. Their life cycle benefits reduce environmental impact during both production and disposal [110]. PLA is used with additives like wood flour or metals to improve strength and broaden its applications. Its recyclability supports circular economy goals. The use of PLA in parklets exemplifies a flexible, ecological, and socially aware approach to urban space design.

**Figure 12:**
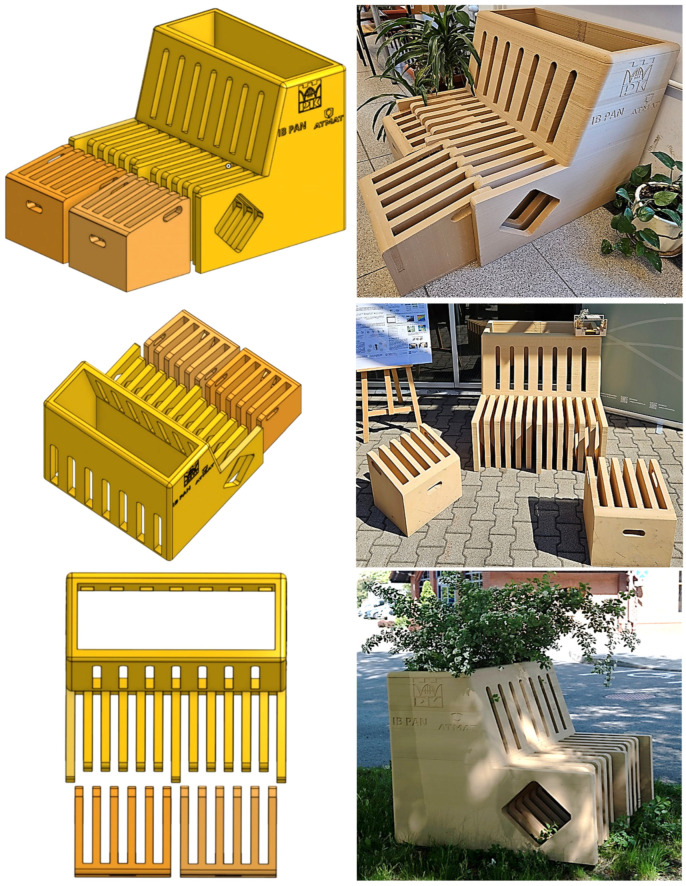
Concept design and real-life implementation of a 3D-printed urban parklet demonstrator. The left panel illustrates the conceptual CAD models of a modular parklet, comprising a main seating structure and detachable stools. The designs feature slotted openings to enhance ergonomics and material efficiency. The right panel presents photographs of the realized parklet demonstrator in various urban and exhibition settings.

Figure 12 presents the design and realization of a functional parklet demonstrator. The design process included CAD modeling and subsequent fabrication by large-format dual-material 3D printing. A key innovation in the fabrication of this demonstrator was the use of dual-head extrusion 3D printing, which allowed for two different PLA-based materials to be used simultaneously within a single print. The inner structural core was printed using PPf filament, chosen for its superior mechanical strength and thermal stability. In contrast, the outer shell was printed using a wood-filled PPWf composite, which provided a warm, tactile finish and enhanced visual appeal suitable for outdoor, user-facing applications. The dual-material strategy offered multiple functional benefits: (1) improved mechanical performance, as the core material contributed to structural integrity while reducing risks of delamination or deformation under load; (2) enhanced surface aesthetics and sustainability, thanks to the outer wood-based PLA, which mimics natural wood grain and is partially biogenic; (3) material optimization, as the more expensive or moisture-sensitive wood composite was used only where necessary (externally), reducing waste and print time. The final demonstrator was printed at full scale and assembled for an outdoor exhibition. The completed unit includes a two-person bench with ribbed seating to improve water runoff and comfort, a part for planter inserts for greenery integration, and detachable stools that can be stored under the main seat or used independently. The successful fabrication of this demonstrator confirms the feasibility of multi-material 3D printing for practical, outdoor applications and illustrates the potential of combining functionally tailored materials for design, comfort, and performance in sustainable urban furniture.

In summary, designed parklets, as modular elements of urban microarchitecture, contribute directly to urban sustainability by enabling the decentralized production of public furniture through large-format 3D printing (modular, scalable design for flexible deployment) using eco-friendly composite materials (biodegradable, renewable materials). Their fabrication via additive manufacturing allows for rapid, site-specific customization with minimal material waste and reduced environmental impact (on-demand local production with reduced transport emissions). The use of biodegradable and bio-based filaments—such as PLA blended with PBAT or reinforced with wood fibers—supports low-carbon material cycles and circular design principles. Additionally, the ability to integrate passive functionalities (e.g., water drainage channels, ventilated surfaces, or green inserts) enhances microclimatic regulation and ecosystem services in dense urban areas (support for walkability, public space equity, and community well-being). As rapidly deployable and recyclable units, 3D-printed parklet offers scalable solutions for sustainable city-making aligned with smart infrastructure and climate-adaptive design.

## 4. Conclusions

This study presented dual-material (PLA/PBAT/wood as an external shell and PLA/PBAT as a structural core), large-scale 3D printing of sustainable, modular urban furniture elements. A dual-extrusion strategy employed two printheads with different nozzle diameters and independently controlled layer heights for outer and internal infill. It preserved the surface aesthetic and ecological appeal of natural materials and the mechanical performance of standard PLA-based composites. Thermal, mechanical, and chemical characterization revealed key distinctions between the materials:

- PPW composites undergo the multistage controlled degradation of lignin and cellulose, which supports stable rheological behavior during extrusion. It confirmed the stability of the materials but also highlighted their potential for post-use processing and degradation. These materials could be effectively recycled or repurposed at the end of their life cycle, contributing to a more sustainable approach and waste management.
- Enhanced compatibility and thermal interaction between PLA and wood fibers were found in samples with balanced architecture, resulting in smooth extrusion, better interlayer adhesion, and no delamination or layer separation under aggressive chemical and mechanical treatments. Suboptimal material ratios resulted in excess moisture retention, poorer fiber–polymer compatibility, and printing artifacts such as surface bubbling or interfacial defects.

Overall, this work demonstrates that multi-material 3D printing with bio-based filaments can serve as a powerful tool for designing more sustainable microarchitectural elements with a high social and ecological impact:

- Three-dimensionally printed wood/PLA materials are biodegradable and derived from renewable sources (e.g., corn starch and wood fibers), reducing the reliance on fossil fuels and lowering carbon footprints compared to traditional petroleum-based plastics; the integration of wood-based biocomposites adds value to lignocellulosic waste or sustainably harvested fibers, encouraging circular material flows;
- Three-dimensional printing minimizes material waste due to additive manufacturing’s precision, while localized fabrication (e.g., printing parklets on-site or near-site) reduces transportation emissions and packaging waste, supporting decentralized and eco-friendly production models;
- Modular, low-cost infrastructure like parklets can be deployed quickly in underserved communities, enhancing public space equity and offering inclusive, shared environments, promoting walkability, social interaction, and access to micro-rest spaces—important in densely populated urban areas;
- Demonstrating sustainable 3D printing in public spaces acts as a visible example of eco-innovation, can serve as a living lab for further research and citizen science, potentially influencing public behavior and educational curricula, and creating a blueprint for how emerging fabrication technologies can directly benefit both people and the planet.

Despite the environmental efficiency of PLA/wood biocomposites, their application in outdoor urban infrastructure presents several inherent material limitations that must be considered:

- Three-dimensional printing, especially with dual nozzles, is time-intensive; it may not be viable for large-scale deployment unless batch processes or modular assembly are optimized.
- While marketed as biodegradable, PLA and its wood-filled variant biodegradation typically require industrial composting facilities with sustained temperatures above 60 °C, specific microbial activity, and controlled humidity; in practice, they may remain intact for years, contributing to long-term microplastic pollution if not properly managed.
- Wood/PLA exhibits hygroscopic tendencies, and this can result in dimensional instability (warping or swelling), surface degradation such as cracking or splintering, and the potential for microbial growth (e.g., mold and mildew), particularly in humid or shaded environments or in colder climates, where absorbed moisture can freeze and expand.
- Both neat PLA and wood-filled PLA are highly susceptible to UV-induced degradation. Extended sunlight exposure leads to discoloration and yellowing, loss of tensile strength and brittleness, and microcrack formation at the surface level.

Therefore, the future directions of the research in this arena lie in long-term real-environment testing of parklet properties and material behaviors, e.g.,

- Monitoring the impact of UV exposure, rainfall, humidity, temperature fluctuations, and freeze–thaw cycles on the structural integrity, surface quality, and development of mold or algae, especially on wood-filled elements;
- Evaluating wear patterns from human contact, friction, and airborne particles (dust and pollutants), particularly in high-use public areas;
- Developing the recycling or reprocessing of dual-material components, including mechanical grinding, filament re-extrusion, or chemical recycling methods.

## Figures and Tables

**Table 1:** Material selection criteria for 3D printing small architectural models.

Factor	PLA 3D870	PBAT Ecoflex	CW630PU
Printability	Minimal warping and high dimensional accuracy; ideal for complex architectural details.	Flexible, ductile, and useful for adding toughness or flexibility to PLA.	Composites can be extruded without nozzle clogging and offer decent layer adhesion.
Mechanical strength	Enhanced resistance and strength compared to standard PLA.	Improves flexibility, toughness, and resistance when blended with PLA.	Adds stiffness and structural integrity to matrices.
Dimensional stability	Dimensional accuracy provided proper cooling/annealing.	Good flexibility; less stability for precision models.	Reduces shrinkage and warping when used in composites.
Heat resistance	Improved thermal resistance.	Lower thermal resistance.	Low heat resistance.
Surface finish/aesthetic appeal	Fine detail and smooth surfaces; critical for an architectural model.	Flexible; used in blends rather than for visual appeal	Texture improves the visual authenticity of prints.
Biodegradability	More environmentally friendly than petroleum-based plastics.	compostable under industrial conditions.	High biodegradability and low environmental impact.
Sustainability	Derived from renewable resources (like corn), contributing to a reduced carbon footprint	Suitable for green applications due to its fully compostable nature.	Natural fibers support eco-conscious manufacturing and sustainability goals.

**Table 2:** Composition of materials used for filament (PPf and PPWf) and samples (PPp and PPWp) production.

Identifier	PLA [% wt.]	PBAT [% wt.]	Wood [% wt.]
PLA	100	-	-
PLA/PBAT (PPf and PPp)	60	40	-
PLA/PBAT/wood (PPWf and PPWp)	48	32	20

**Table 3:** Settings for the granulation unit and extruder.

Granulation Unit EUP 50 (4 mm Perforated Plate)					
Base temp	Inlet temp	Head temp	Water bath temp	Melt pressure	Blade speed
170 °C	175 °C	170 °C	60 °C	10 bar	78 rpm
**Extruder TSK 30**					
Heating Zone 4	Zone 3	Zone 2	Zone 1	Throughput	Screw speed
204 °C	181 °C	160 °C	154 °C	2 kg h−1	500 rpm

**Table 4:** Printing parameters for external outlines and infill regions.

Feature	External Outline	Infill Region (Enhanced Layers)
Layer height	0.5 mm; 0.8 mm	0.5–2.4 mm
Flow rate	100%	120–130%
Printing speed	25 mm s^−1^	35–60 mm s^−1^
Infill pattern	Rectilinear (45°/−45°)	Rectilinear (45°/−45°)

**Table 5:** Sample PLA/PBAT/wood (PPWp) and PLA/PBAT (PPp) with the composition presented in Table 2, as well as a layer geometry (samples 1–4) of external outlines and infill regions.

External Outline			Infill Region				Outline to Infill Ratio
Composition	Layer (mm)		Composition	Nozzle (mm)	Layer (mm)	Infill (%)	
PPWp1	0.5		PPp1	1.0	0.5	25	1:1
PPWp2	0.5		PPp2	1.0	1.0	25	1:1
PPWp3	0.5		PPp3	1.4	1.5	25	1:3
PPWp4	0.8		PPp4	1.4	2.4	25	1:3

**Table 6:** Printing parameters for PLA/PBAT/wood (PPW) filament and reference filament (PLA Fiberlogy, Fiberlab Ltd. Brzezie, Poland).

Parameter	PPW	Reference (PLA Fiberlogy)
Printhead	Main	Auxiliary
Strategy	Outline—2 layers	Infill
Single layer width	2.00 mm	4.50 mm
Layer height	1.00 mm	1.50 mm
Flow rate	100%	100%
Temperature (nozzle/build plate)	230 °C/60 °C	220 °C/50 °C
Processing speed (the first layer)	20 mm s^−1^	20 mm s^−1^
Processing speed	30 mm s^−1^	80 mm s^−1^

**Table 7:** Tensile strength and elongation values for dual-material 3D printed samples with varying outer shell and core configurations. Each sample designation corresponds to a specific combination of wood-filled PLA (PPWp) and unfilled PLA/PBAT (PPp) filaments. Values represent the mean ± standard deviation from 5 replicate tests.

Sample Designation	Tensile Strength (N mm^−2^)	Elongation (mm)
PPWp1/PPp1	6.5 ± 0.14	3.7 ± 0.17
PPWpp2/PPp2	6.8 ± 0.14	3.7 ± 0.19
PPWp3/PPp3	7.9 ± 0.14	3.5 ± 0.21
PPWp4/PPp4	7.7 ± 0.49	3.4 ± 0.27

## Data Availability

The original contributions presented in this study are included in the article. Further inquiries can be directed to the corresponding author.

## References

[1] Zheng J., Chen A., Zheng W., Zhou X., Bai B., Wu J., Ling W., Ma H., Wang W. (2020). Effectiveness analysis of resources consumption, environmental impact and production efficiency in traditional manufacturing using new technologies: Case from sand casting. Energy Convers. Manag..

[2] Gao X., Qi S., Kuang X., Su Y., Li J., Wang D. (2021). Fused filament fabrication of polymer materials: A review of interlayer bond. Addit. Manuf..

[3] Marczyk J., Ziejewska C., Korniejenko K., Łach M., Marzec W., Góra M., Dziura P., Sprince A., Szechyńska-Hebda M., Hebda M. (2022). Properties of 3D printed concrete–geopolymer hybrids reinforced with aramid roving. Materials.

[4] Leschok M., Piccioni V., Lydon G., Seshadri B., Schlueter A., Gramazio F., Kohler M., Dillenburger B. (2024). Thermal and manufacturing properties of hollow-core 3D-printed elements for lightweight facades. Dev. Built Environ..

[5] Li A., Challapalli A., Li G. (2019). 4D printing of recyclable lightweight architectures using high recovery stress shape memory polymer. Sci. Rep..

[6] Szechyńska-Hebda M., Hebda M., Doğan-Sağlamtimur N., Lin W.-T. (2024). Let’s print an ecology in 3D (and 4D). Materials.

[7] Eco-Efficiency Assessment of Bioplastics Production Systems and End-of-Life Options. https://www.mdpi.com/2071-1050/10/4/952.

[8] Valino A.D., Dizon J.R.C., Espera A.H., Chen Q., Messman J., Advincula R.C. (2019). Advances in 3D printing of thermoplastic polymer composites and nanocomposites. Prog. Polym. Sci..

[9] Zhao X., Wang Y., Chen X., Yu X., Li W., Zhang S., Meng X., Zhao Z.-M., Dong T., Anderson A. (2023). Sustainable bioplastics derived from renewable natural resources for food packaging. Matter.

[10] Coppola G., Gaudio M.T., Lopresto C.G., Calabro V., Curcio S., Chakraborty S. (2021). Bioplastic from renewable biomass: A facile solution for a greener environment. Earth Syst. Environ..

[11] Zhang F., Sun Y., Li J., Su H., Zhu Z., Yan B., Cheng Z., Chen G. (2022). Pyrolysis of 3D printed polylactic acid waste: A kinetic study via TG-FTIR/GC-MS analysis. J. Anal. Appl. Pyrolysis.

[12] Sabalina A., Platnieks O., Gaidukova G., Aunins A., Eiduks T.V., Gaidukovs S. (2025). Thermomechanical and mechanical analysis of polylactic acid/polyhydroxyalkanoate/poly(butylene succinate-co-adipate) binary and ternary blends. RSC Adv..

[13] dos Santos Filho E.A., Luna C.B.B., da Silva Barbosa Ferreira E., Pinto G.M., Andrade R.J.E., Fechine G.J.M., Araújo E.M. (2025). Enhancing PLA/ABS blends compatibility: A comparative study with SAN-Epoxy and SAN-MA. Polym. Adv. Technol..

[14] Travieso-Rodriguez J.A., Jerez-Mesa R., Llumà J., Gomez-Gras G., Casadesus O. (2020). Comparative study of the flexural properties of ABS, PLA and a PLA-wood composite manufactured through fused filament fabrication. Rapid Prototyp. J..

[15] Bermudez D., Quiñonez P.A., Vasquez E.J., Carrete I.A., Word T.J., Roberson D.A. (2021). A Comparison of the physical properties of two commercial 3D printing PLA grades. Virtual Phys. Prototyp..

[16] Le Duigou A., Correa D., Ueda M., Matsuzaki R., Castro M. (2020). A review of 3D and 4D printing of natural fibre biocomposites. Mater. Des..

[17] Siddiqui M.A.S., Rabbi M.S., Ahmed R.U., Billah M.M. (2024). Biodegradable natural polymers and fibers for 3D printing: A holistic perspective on processing, characterization, and advanced applications. Clean. Mater..

[18] Blok L.G., Longana M.L., Yu H., Woods B.K.S. (2018). An investigation into 3D printing of fibre reinforced thermoplastic composites. Addit. Manuf..

[19] Góra M. (2025). Methods of Using Solid Reinforcement Additives in the 3D Printing Process. Ph.D. Thesis.

[20] Gauss C., Pickering K.L., Graupner N., Müssig J. (2023). 3D-printed polylactide composites reinforced with short lyocell fibres—Enhanced mechanical properties based on bio-inspired fibre fibrillation and post-print annealing. Addit. Manuf..

[21] Bhandari S., Lopez-Anido R.A., Gardner D.J. (2019). Enhancing the interlayer tensile strength of 3D printed short carbon fiber reinforced PETG and PLA composites via annealing. Addit. Manuf..

[22] Jamadar I.M., Kamate P., Samal P.K. (2024). Evaluation of fatigue characteristics of 3D printed/composites reinforced with carbon fiber using design of experiments. Polym. Compos..

[23] Charca S., Jiao-Wang L., Loya J.A., Martínez M.A., Santiuste C. (2024). High cycle fatigue life analysis of unidirectional flax/PLA composites through infrared thermography. Compos. Struct..

[24] Olcun S., Ibrahim Y., Isaacs C., Karam M., Elkholy A., Kempers R. (2023). Thermal conductivity of 3D-printed continuous pitch carbon fiber composites. Addit. Manuf. Lett..

[25] Chen Y., Ye L., Kinloch A.J., Zhang Y.X. (2022). 3D printed carbon-fibre reinforced composite lattice structures with good thermal-dimensional stability. Compos. Sci. Technol..

[26] Okubo K., Fujii T., Thostenson E.T. (2009). Multi-scale hybrid biocomposite: Processing and mechanical characterization of bamboo fiber reinforced PLA with microfibrillated cellulose. Compos. Part Appl. Sci. Manuf..

[27] Raj G., Balnois E., Helias M.-A., Baley C., Grohens Y. (2012). Measuring adhesion forces between model polysaccharide films and PLA bead to mimic molecular interactions in flax/PLA biocomposite. J. Mater. Sci..

[28] Bhagia S., Bornani K., Agrawal R., Satlewal A., Ďurkovič J., Lagaňa R., Bhagia M., Yoo C.G., Zhao X., Kunc V. (2021). Critical review of FDM 3D printing of PLA biocomposites filled with biomass resources, characterization, biodegradability, upcycling and opportunities for biorefineries. Appl. Mater. Today.

[29] Park Y.-E., Lee S. (2024). Characterization of PLA/LW-PLA composite materials manufactured by Dual-Nozzle FDM 3D-printing processes. Polymers.

[30] Vinod A., Tengsuthiwat J., Vijay R., Sanjay M.R., Siengchin S. (2024). Advancing additive manufacturing: 3D-printing of hybrid natural fiber sandwich (Nona/Soy-PLA) composites through filament extrusion and its effect on thermomechanical properties. Polym. Compos..

[31] Li M., Lei W., Yu W. (2024). FDM 3D Printing and properties of WF/PBAT/PLA composites. Molecules.

[32] Cao A., Wan D., Gao C., Elverum C.W. (2024). A novel method of fabricating designable polylactic acid (PLA)/thermoplastic polyurethane (TPU) composite filaments and structures by material extrusion additive manufacturing. J. Manuf. Process..

[33] Tadi S.P., Mamilla R.S. (2025). Fabrication of SS 316L particle-infilled PLA composite filaments from cast-off bi-material extrudates for 3D printing applications. Waste Manag..

[34] dos Santos A.L., de Souza F.C.R., Martins da Costa J.C., Gonçalves D.A., Passos R.R., Pocrifka L.A. (2024). Development and characterization of 3D-Printed PLA/Exfoliated graphite composites for enhanced electrochemical performance in energy storage applications. Polymers.

[35] Cho J.-Y., Oh Y.-C., Shin S.-C., Lee S.-K., Seo H.-S., Lee S.-E. (2024). Fusedly Deposited frequency-selective composites fabricated by a dual-nozzle 3D printing as microwave filter. Polymers.

[36] Tirado-Garcia I., Garcia-Gonzalez D., Garzon-Hernandez S., Rusinek A., Robles G., Martinez-Tarifa J.M., Arias A. (2021). Conductive 3D printed PLA composites: On the interplay of mechanical, electrical and thermal behaviours. Compos. Struct..

[37] Kumar S., Singh R., Singh M. (2022). Multi-material 3D printed PLA/PA6-TiO_2_ composite matrix: Rheological, thermal, tensile, morphological and 4D capabilities. Adv. Mater. Process. Technol..

[38] Bledzki A.K., Gassan J. (1999). Composites reinforced with cellulose based fibres. Prog. Polym. Sci..

[39] Gholampour A., Ozbakkaloglu T. (2020). A review of natural fiber composites: Properties, modification and processing techniques, characterization, applications. J. Mater. Sci..

[40] Bax B., Müssig J. (2008). Impact and tensile properties of PLA/Cordenka and PLA/flax composites. Compos. Sci. Technol..

[41] Effect of Process Parameters on Void Distribution, Volume Fraction, and Sphericity Within the Bead Microstructure of Large-Area Additive Manufacturing Polymer Composites. https://www.mdpi.com/2073-4360/14/23/5107.

[42] Schirmeister C.G., Hees T., Licht E.H., Mülhaupt R. (2019). 3D printing of high density polyethylene by fused filament fabrication. Addit. Manuf..

[43] Jiang L., Wolcott M.P., Zhang J. (2006). Study of biodegradable polylactide/poly(butylene adipate-co-terephthalate) blends. Biomacromolecules.

[44] Gu S.-Y., Zhang K., Ren J., Zhan H. (2008). Melt rheology of polylactide/poly(butylene adipate-*co*-terephthalate) blends. Carbohydr. Polym..

[45] Lyu Y., Chen Y., Lin Z., Zhang J., Shi X. (2020). Manipulating phase structure of biodegradable PLA/PBAT system: Effects on dynamic rheological responses and 3D printing. Compos. Sci. Technol..

[46] Yu W., Li M., Lei W., Chen Y. (2024). FDM 3D printing and properties of PBAT/PLA blends. Polymers.

[47] Guo R., Ren Z., Bi H., Song Y., Xu M. (2018). Effect of toughening agents on the properties of poplar wood flour/poly (lactic acid) composites fabricated with Fused Deposition Modeling. Eur. Polym. J..

[48] Dalu M., Temiz A., Altuntaş E., Demirel G.K., Aslan M. (2019). Characterization of tanalith E treated wood flour filled polylactic acid composites. Polym. Test..

[49] Yu W., Li M., Lei W., Pu Y., Sun K., Ma Y. (2022). Effects of Wood Flour (WF) pretreatment and the addition of a toughening agent on the properties of FDM 3D-printed WF/Poly(lactic acid) biocomposites. Molecules.

[50] ISO 527-2:2025 Plastics—Determination of Tensile Properties. https://www.iso.org/standard/85822.html.

[51] ISO 527-1:2019 Plastics—Determination of Tensile Properties, Part 1: General Principles. https://www.iso.org/standard/75824.html.

[52] Szechyńska-Hebda M., Hebda M., Mirek M., Miernik K. (2016). Cold-induced changes in cell wall stability determine the resistance of winter triticale to fungal pathogen Microdochium nivale. J. Therm. Anal. Calorim..

[53] Szechyńska-Hebda M., Hebda M., Mierzwiński D., Kuczyńska P., Mirek M., Wędzony M., van Lammeren A., Karpiński S. (2013). Effect of cold-induced changes in physical and chemical leaf properties on the resistance of winter triticale (×Triticosecale) to the fungal pathogen Microdochium nivale. Plant Pathol..

[54] Szechyńska-Hebda M., Czarnocka W., Hebda M., Karpiński S. (2016). PAD4, LSD1 and EDS1 regulate drought tolerance, plant biomass production, and cell wall properties. Plant Cell Rep..

[55] Sun C., Li C., Tan H., Zhang Y. (2019). Synergistic effects of wood fiber and polylactic acid during co-pyrolysis using TG-FTIR-MS and Py-GC/MS. Energy Convers. Manag..

[56] Sedničková M., Pekařová S., Kucharczyk P., Bočkaj J., Janigová I., Kleinová A., Jochec-Mošková D., Omaníková L., Perďochová D., Koutný M. (2018). Changes of physical properties of PLA-based blends during early stage of biodegradation in compost. Int. J. Biol. Macromol..

[57] Nandiyanto A.B.D., Ragadhita R., Fiandini M. (2023). Interpretation of Fourier Transform Infrared Spectra (FTIR): A practical approach in the polymer/plastic thermal decomposition. Indones. J. Sci. Technol..

[58] Müller G., Schöpper C., Vos H., Kharazipour A., Polle A. (2009). FTIR-ATR spectroscopic analyses of changes in wood properties during particle- and fibreboard production of hard- and softwood trees. BioRes Sci..

[59] Ayatollahi M.R., Nabavi-Kivi A., Bahrami B., Yazid Yahya M., Khosravani M.R. (2020). The influence of in-plane raster angle on tensile and fracture strengths of 3D-printed PLA specimens. Eng. Fract. Mech..

[60] Stoia D.I., Linul E. (2024). Tensile, flexural and fracture properties of MEX-printed PLA-based composites. Theor. Appl. Fract. Mech..

[61] Mazur K.E., Borucka A., Kaczor P., Gądek S., Bogucki R., Mirzewiński D., Kuciel S. (2022). Mechanical, Thermal and microstructural characteristic of 3D printed polylactide composites with natural fibers: Wood, bamboo and cork. J. Polym. Environ..

[62] Phan N.-T., Auslender F., Gril J., Moutou Pitti R. (2024). Effects of cellulose fibril cross-linking on the mechanical behavior of wood at different scales. Wood Sci. Technol..

[63] Li Z., Lin Y., Cheng H., Qian S., Fang X. (2025). Synergistic effects of thermoplastic starch and nanofibrillated cellulose on the mechanical, thermal, and micromorphological properties of polylactic acid composites. Polym. Compos..

[64] Shamsudin Z., Dom A.H.M., Razak M.A.A., Mesri M., Mulyadi (2024). Analysis on the effect of elevated loading of reclaimed spent bleach earth on physico-mechanical properties of PLA polymer composite in single screw extrusion process. J. Adv. Res. Appl. Mech..

[65] Yang X., Steck J., Yang J., Wang Y., Suo Z. (2021). Degradable plastics are vulnerable to cracks. Engineering.

[66] Brüster B., Martin A., Bardon J., Koutsawa Y., Bernstorff S., Raquez J.-M., André S., Dubois P., Addiego F. (2018). In situ multiscale study of deformation heterogeneities in polylactide-based materials upon drawing: Influence of initial crystallinity and plasticization. J. Polym. Sci. Part B Polym. Phys..

[67] Radzif A.A., Chai A.B., Ch’ng S.Y., Tshai K.Y. (2024). Study of the chemical endurance of particulate reinforced thermoplastic composites. Int. J. Nanoelectron. Mater..

[68] Zhao X., Yu J., Liang X., Huang Z., Li J., Peng S. (2023). Crystallization behaviors regulations and mechanical performances enhancement approaches of polylactic acid (PLA) biodegradable materials modified by organic nucleating agents. Int. J. Biol. Macromol..

[69] Li Y., Qiu S., Sun J., Ren Y., Wang S., Wang X., Wang W., Li H., Fei B., Gu X. (2022). A new strategy to prepare fully bio-based poly(lactic acid) composite with high flame retardancy, UV resistance, and rapid degradation in soil. Chem. Eng. J..

[70] Wu Y., Wu J., Yang F., Tang C., Huang Q. (2019). Effect of H_2_O_2_ bleaching treatment on the properties of finished transparent wood. Polymers.

[71] Lu D., Xiong X., Lu G., Gui C., Pang X. (2023). Effects of NaOH/H_2_O_2_/Na_2_SiO_3_ bleaching pretreatment method on wood dyeing properties. Coatings.

[72] Rosu L., Varganici C., Mustata F., Rosu D., Rosca I., Rusu T. (2020). Epoxy Coatings Based on Modified Vegetable Oils for Wood Surface Protection against Fungal Degradation. ACS Appl. Mater. Interfaces.

[73] Rowell R.M. (2006). Chemical modification of wood: A short review. Wood Mater. Sci. Eng..

[74] Plaza N.Z., Pingali S.V., Ibach R.E. (2022). Nanostructural changes correlated to decay resistance of chemically modified wood fibers. Fibers.

[75] Papadopoulos A.N., Bikiaris D.N., Mitropoulos A.C., Kyzas G.Z. (2019). Nanomaterials and chemical modifications for enhanced key wood properties: A review. Nanomaterials.

[76] Shao Z., Kumagai S., Kameda T., Saito Y., Yoshioka T. (2023). Effects of heating rate and temperature on product distribution of poly-lactic acid and poly-3-hydroxybutyrate-co-3-hydroxyhexanoate. J. Mater. Cycles Waste Manag..

[77] Adachi W., Kumagai S., Shao Z., Saito Y., Yoshioka T. (2024). Selective recovery of pyrolyzates of biodegradable (PLA, PHBH) and common plastics (HDPE, PP, PS) during co-pyrolysis under slow heating. Sci. Rep..

[78] Zheng Y., Xu S., Yu C., Pan P. (2022). Stereocomplexed materials of chiral polymers tuned by crystallization: A case study on poly(lactic acid). Acc. Mater. Res..

[79] Grabowska B., Skowron M., Kaczmarska K. (2023). Polylactide used as filment in 3d printing—Part 2: TG-DTG, DSC and drift investigations. J. Cast. Mater. Eng..

[80] Hebda M., Laska M., Szechyńska-Hebda M. (2013). Application of a device used for observation of controlled thermal processes in a furnace. J. Therm. Anal. Calorim..

[81] Grigsby W.J., Torayno D., Gaugler M., Luedtke J., Krause A. (2022). Chemical imaging of the polylactic acid—Wood adhesion interface of bonded veneer products. Fibers.

[82] Pop N., Mogoş A.M., Vlase G., Vlase T., Doca N. (2013). Theoretic analysis and experimental evidence for relationships between the derivative thermogravimetric curves and the Gramm–Schmidt profiles. J. Therm. Anal. Calorim..

[83] Tan L., Shi R., Ji Q., Wang B., Quan F., Xia Y. (2017). Effect of Na^+^ and Ca^2+^ on the thermal degradation of carboxymethylcellulose in air. Polym. Polym. Compos..

[84] Zou H., Yi C., Wang L., Liu H., Xu W. (2009). Thermal degradation of poly(lactic acid) measured by thermogravimetry coupled to Fourier transform infrared spectroscopy. J. Therm. Anal. Calorim..

[85] Shen D.K., Gu S. (2009). The mechanism for thermal decomposition of cellulose and its main products. Bioresour. Technol..

[86] Hong T., Yin J.-Y., Nie S.-P., Xie M.-Y. (2021). Applications of infrared spectroscopy in polysaccharide structural analysis: Progress, challenge and perspective. Food Chem. X.

[87] Lahlali R., Song T., Chu M., Yu F., Kumar S., Karunakaran C., Peng G. (2017). Evaluating changes in cell-wall components associated with clubroot resistance using fourier transform infrared spectroscopy and RT-PCR. Int. J. Mol. Sci..

[88] Mukherjee T., Tobin M.J., Puskar L., Sani M.-A., Kao N., Gupta R.K., Pannirselvam M., Quazi N., Bhattacharya S. (2017). Chemically imaging the interaction of acetylated nanocrystalline cellulose (NCC) with a polylactic acid (PLA) polymer matrix. Cellulose.

[89] Mahmud S., Long Y., Wang J., Dai J., Zhang R., Zhu J. (2020). Waste cellulose fibers reinforced polylactide toughened by direct blending of epoxidized soybean oil. Fibers Polym..

[90] Pornbencha K., Boonmalert T., Seubsai A., Dittanet P. (2021). Synthesis of polylactic acid/cellulose composite extracted from pineapple leaves. Key Eng. Mater..

[91] Kumar S., Dang R., Manna A., Dhiman N.K., Sharma S., Dwivedi S.P., Kumar A., Li C., Tag-Eldin E.M., Abbas M. (2023). Optimization of chemical treatment process parameters for enhancement of mechanical properties of Kenaf fiber-reinforced polylactic acid composites: A comparative study of mechanical, morphological and microstructural analysis. J. Mater. Res. Technol..

[92] Cruz Fabian D.R., Durpekova S., Dusankova M., Cisar J., Drohsler P., Elich O., Borkova M., Cechmankova J., Sedlarik V. (2023). Renewable poly(lactic acid)lignocellulose biocomposites for the enhancement of the water retention capacity of the soil. Polymers.

[93] Gwon J.G., Lee S.Y., Doh G.H., Kim J.H. (2010). Characterization of chemically modified wood fibers using FTIR spectroscopy for biocomposites. J. Appl. Polym. Sci..

[94] Partini M., Pantani R. (2007). Determination of crystallinity of an aliphatic polyester by FTIR spectroscopy. Polym. Bull..

[95] Yang S., Liu Z., Liu Y., Jiao Y. (2015). Effect of molecular weight on conformational changes of PEO: An infrared spectroscopic analysis. J. Mater. Sci..

[96] Poletto M., Zattera A.J., Santana R.M.C. (2012). Structural differences between wood species: Evidence from chemical composition, FTIR spectroscopy, and thermogravimetric analysis. J. Appl. Polym. Sci..

[97] Niu S., Zhou Y., Yu H., Lu C., Han K. (2017). Investigation on thermal degradation properties of oleic acid and its methyl and ethyl esters through TG-FTIR. Energy Convers. Manag..

[98] Gong L., Pan Y., Cui L., Zhang X. (2024). Atomic insights into the thermal degradation of polyethylene terephthalate combining STA-FTIR and DFT methods. Fuel.

[99] Oladapo B.I., Ismail S.O., Zahedi M., Khan A., Usman H. (2020). 3D printing and morphological characterisation of polymeric composite scaffolds. Eng. Struct..

[100] Vidakis N., Petousis M., Tzounis L., Maniadi A., Velidakis E., Mountakis N., Papageorgiou D., Liebscher M., Mechtcherine V. (2021). Sustainable additive manufacturing: Mechanical response of polypropylene over multiple recycling processes. Sustainability.

[101] Pricop B., Sava Ș.D., Lohan N.-M., Bujoreanu L.-G. (2022). DMA Investigation of the factors influencing the glass transition in 3D printed specimens of shape memory recycled PET. Polymers.

[102] Zhou C., Li H., Zhang Y., Xue F., Huang S., Wen H., Li J., de Christiansen J.C., Yu D., Wu Z. (2015). Deformation and structure evolution of glassy poly(lactic acid) below the glass transition temperature. CrystEngComm.

[103] Ohtani Y., Okumura K., Kawaguchi A. (2003). Crystallization behavior of amorphous poly(l-Lactide). J. Macromol. Sci. Part B.

[104] Masanet E., Heeren N., Kagawa S., Cullen J., Lifset R., Wood R. (2021). Material efficiency for climate change mitigation. J. Ind. Ecol..

[105] Tupenaite L., Kanapeckiene L., Naimaviciene J., Kaklauskas A., Gecys T. (2023). Timber construction as a solution to climate change: A systematic literature review. Buildings.

[106] Korkmaz E. Parklets As Public Space. Proceedings of the International Conference of Contemporary Affairs in Architecture and Urbanism-ICCAUA.

[107] Stevens Q., Leorke D., Thai H.M.H., Innocent T., Tolentino C. (2024). Playful, portable, pliable interventions into street spaces: Deploying a ‘playful parklet’ across Melbourne’s suburbs. J. Urban Des..

[108] Iždinský J., Reinprecht L., Vidholdová Z. (2021). Particleboards from recycled pallets. Forests.

[109] Negawo T.A., Polat Y., Kilic A. (2021). Effect of compatibilizer and fiber loading on ensete fiber-reinforced HDPE green composites: Physical, mechanical, and morphological properties. Compos. Sci. Technol..

[110] Beigbeder J., Soccalingame L., Perrin D., Bénézet J.-C., Bergeret A. (2019). How to manage biocomposites wastes end of life? A life cycle assessment approach (LCA) focused on polypropylene (PP)/wood flour and polylactic acid (PLA)/flax fibres biocomposites. Waste Manag..

